# K^+^‐Triggered Defect Engineering and Proton‐Coupled Storage in V_2_O_5_·nH_2_O for Advanced Zn‐Ion Thin‐Film and Microbatteries

**DOI:** 10.1002/advs.75851

**Published:** 2026-05-29

**Authors:** Jingli Luo, Sanat Nalini Paltasingh, Bastola Narayan, Yijia Zhu, Debashish Das, Shuhui Li, Firoz Alam, Subhra R. Pattanayak, Sijin Liu, Tharangattu N. Narayanan, Georgios Nikiforidis, Gopinathan Sankar, Ivan P. Parkin, Saroj Kumar Nayak, Buddha Deka Boruah

**Affiliations:** ^1^ Institute for Materials Discovery University College London London UK; ^2^ School of Basic Sciences Indian Institute of Technology Bhubaneswar Khordha Odisha India; ^3^ Department of Mechanical Engineering University of Bath Bath UK; ^4^ Department of Chemistry University College London London UK; ^5^ Department of Electronic and Electrical Engineering University College London London UK; ^6^ Tata Institute of Fundamental Research Hyderabad Hyderabad India

**Keywords:** defect‐mediated charge transport, hydrated vanadium oxide, oxygen vacancy engineering, potassium pre‐intercalation, proton‐Zn^2^^+^ co‐storage, thin‐film batteries, zinc‐ion microbatteries

## Abstract

The increasing demand for wearable electronics, point‐of‐care diagnostics, and integrated microsystems necessitates thin‐film and microbatteries that combine high energy density, fast kinetics, and intrinsic safety. In this work, we develop Zn‐based thin‐film batteries (Zn‐TFBs) and microbatteries (Zn‐MBs) using K^+^‐pre‐intercalated V_2_O_5_·nH_2_O cathodes and reveal a fundamentally distinct charge‐storage mechanism. Contrary to the conventional paradigm where metal‐ion pre‐intercalation merely enlarges interlayer spacing, K^+^ incorporation induces interlayer contraction accompanied by substantial oxygen‐vacancy generation and mixed‐valence (V^4^^+^/V^5^^+^) formation. These coupled lattice and electronic modulations activate proton‐dominated transport pathways, enabling cooperative H^+^/Zn^2^^+^ co‐storage and markedly accelerated reaction kinetics. Density functional theory calculations further confirm that the enhanced electrochemical behavior cannot be explained by interlayer expansion alone, but originates from defect‐mediated proton conduction and vacancy‐stabilized redox centers. Benefiting from this defect‐engineered proton‐Zn^2^^+^ synergistic storage, the K^+^‐modified V_2_O_5_·nH_2_O cathode delivers an areal capacity of 200.9 µAh cm^−^^2^ and an areal energy of 150 µWh cm^−^^2^ at 50 µA cm^−^^2^ in Zn‐TFBs, together with a high areal capacity of 49 µAh cm^−^^2^ in Zn‐MBs. This study establishes K^+^‐triggered defect and valence‐state engineering as a powerful strategy to regulate proton‐coupled charge storage in hydrated vanadium oxides, opening a viable pathway toward high‐energy Zn‐based energy‐storage systems.

## Graphical abstract

K^+^ pre‐intercalation in V_2_O_5_·nH_2_O induces interlayer contraction, oxygen‐vacancy formation, and mixed‐valence states, activating defect‐mediated proton‐dominated transport. This enables cooperative H^+^/Zn^2^^+^ storage with enhanced kinetics and stability. The resulting Zn‐based thin‐film and microbatteries deliver high areal energy and capacity, offering a promising strategy for scalable, high‐performance on‐chip energy storage systems.

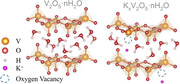

## 1 Introduction

The growing demand for portable, flexible, and intelligent electronics has driven a global search for safe, miniaturized, and high‐performance energy storage systems. From the first large‐scale electronic equipment to today's micro‐sized pet locators, wearable biosensors, implantable medical devices, smart cards, and RFID tags, electronic technology has evolved rapidly toward wearability and integration [1, 2]. To reliably power these devices, compatible secondary batteries—particularly bendable thin‐film batteries (TFBs) and miniaturized interdigital microbatteries (MBs)—are urgently required [3, 4]. Lithium‐ion batteries (LIBs) have dominated the secondary battery field for decades due to their high operating potential, excellent energy density, and long cycle life [5]. However, their safety concerns, especially the risk of thermal runaway and explosion, as well as the limited lithium resource reserves and high production cost, hinder their broader application in next‐generation wearable electronics and PoC devices [6]. To overcome these challenges, alternative rechargeable metal‐ion systems (e.g., Na^+^, K^+^, Mg^2^^+^, Zn^2^^+^) have been investigated [7]. Among them, zinc‐ion batteries (ZIBs) stand out due to their abundance, safety, low cost, aqueous electrolyte compatibility enabled by a suitable redox potential (–0.76 V vs. SHE), and the high theoretical capacity (820 mAh g^−1^, 5855 mAh cm^−^^3^) of metallic Zn anodes [8, 9]. The performance of ZIBs is highly dependent on cathode design. Various classes of cathode materials have been explored, including vanadium‐based oxides, manganese‐based oxides, organic compounds, and Prussian blue analogues (PBAs) [6, 10, 11, 12]. Manganese‐based cathodes suffer from poor cycling stability due to Mn^3^^+^ disproportionation and Mn^2^^+^ dissolution [10]. Organic cathodes face challenges of low conductivity, side reactions, and dissolution in aqueous electrolytes [11, 13]. PBAs are limited by single‐atom redox chemistry and inefficient active site utilization, resulting in specific capacities below 100 mAh g^−^^1^ [13]. In contrast, vanadium‐based oxides stand out due to their multivalent states (+ 5, + 4, + 3, + 2), which provide multiple redox pathways, high reversible capacity (> 400 mAh g^−^^1^), and structural stability during Zn^2^^+^ insertion/extraction [2, 14, 15]. Vanadium pentoxide (V_2_O_5_), in particular, offers a layered structure, cost‐effectiveness, and well‐established synthesis strategies [16, 17].

Despite these advantages, Zn^2^^+^ intercalation in V_2_O_5_ is hindered by two factors: (i) the large hydrated radius of Zn^2^^+^ compared with Li^+^, which causes significant steric resistance, and (ii) strong electrostatic interactions arising from its divalent charge, leading to sluggish ion diffusion and structural instability [7, 13, 18]. Several strategies have been developed to address these issues. Electrolyte engineering, such as employing Zn(CF_3_SO_3_)_2_ instead of ZnSO_4_, reduces the hydrated radius of Zn^2^^+^ and improves ion mobility [19]. Structural water intercalation broadens interlayer spacing in V_2_O_5_ xerogels, which weakens electrostatic interactions and enhances Zn^2^^+^ diffusion kinetics [20, 21]. Pre‐insertion of metal ions (Li^+^, Na^+^, K^+^, Ni^2^^+^, Zn^2^^+^) into the V_2_O_5_ lattice can also stabilize the layered framework, broaden diffusion pathways, and induce oxygen vacancies that further facilitate ion transport and electronic conductivity through mixed‐valence V^4^^+^/V^5^^+^ states [22, 23, 24, 25, 26, 27, 28]. These oxygen vacancies additionally provide abundant active sites, buffer structural strain, and improve cycling stability [22, 26]. Previous studies have suggested that the enhanced capacity of V_2_O_5_ cathodes after cation pre‐intercalation primarily arises from interlayer expansion, which facilitates Zn^2^^+^ diffusion kinetics. It is still unclear whether interlayer expansion alone accounts for the observed improvements in charge storage, or if cation intercalation always leads to expansion of the V_2_O_5_ interlayer spacing, as reported in many studies [29, 30]. Moreover, it remains an open question whether such expansion is strictly necessary to achieve capacity enhancement [29, 30]. Therefore, gaining an in‐depth understanding of the underlying mechanisms is essential for achieving capacity improvements.This is particularly critical for TFBs and MBs, where enhancing the capacity of cathode materials is crucial due to the limited device footprint. Moreover, improving the intrinsic conductivity of these materials becomes even more important, as electrodes in such systems typically operate without binders or conductive additives.

In this work, we reveal an unexpected and fundamentally new charge‐storage paradigm in K^+^‐pre‐intercalated hydrated V_2_O_5_ thin‐film cathodes for Zn‐based TFBs (Zn‐TFBs) and MBs (Zn‐MBs). Contrary to the prevailing belief that alkali‐metal pre‐intercalation enhances performance by expanding interlayer spacing, K^+^ incorporation in hydrated V_2_O_5_ induces an interlayer contraction while simultaneously triggering a cascade of beneficial electronic effects, including the formation of abundant oxygen vacancies, stabilization of mixed‐valence V^4^^+^/V^5^^+^ redox centers, and a dramatic enhancement in electronic conductivity. Through a combination of advanced spectroscopy, electrochemical analysis, and density‐functional‐theory modelling, we demonstrate that these defect‐driven features unlock a highly efficient proton‐Zn^2^^+^ co‐storage mechanism, enabling rapid ion transport and exceptional charge utilization even within the contracted lattice framework. As a result, the K^+^‐engineered V_2_O_5_·nH_2_O cathodes deliver substantially higher capacities than their pristine counterparts, overturning the conventional interlayer‐expansion paradigm and establishing defect‐mediated ion transport as a powerful new design strategy for safe, high‐rate Zn‐TFBs and Zn‐MBs. This study opens a transformative pathway toward ultra‐compact, high‐performance energy‐storage systems for next‐generation wearable, implantable, and on‐chip electronics.

## 2 Results and Discussion

**FIGURE 1:**
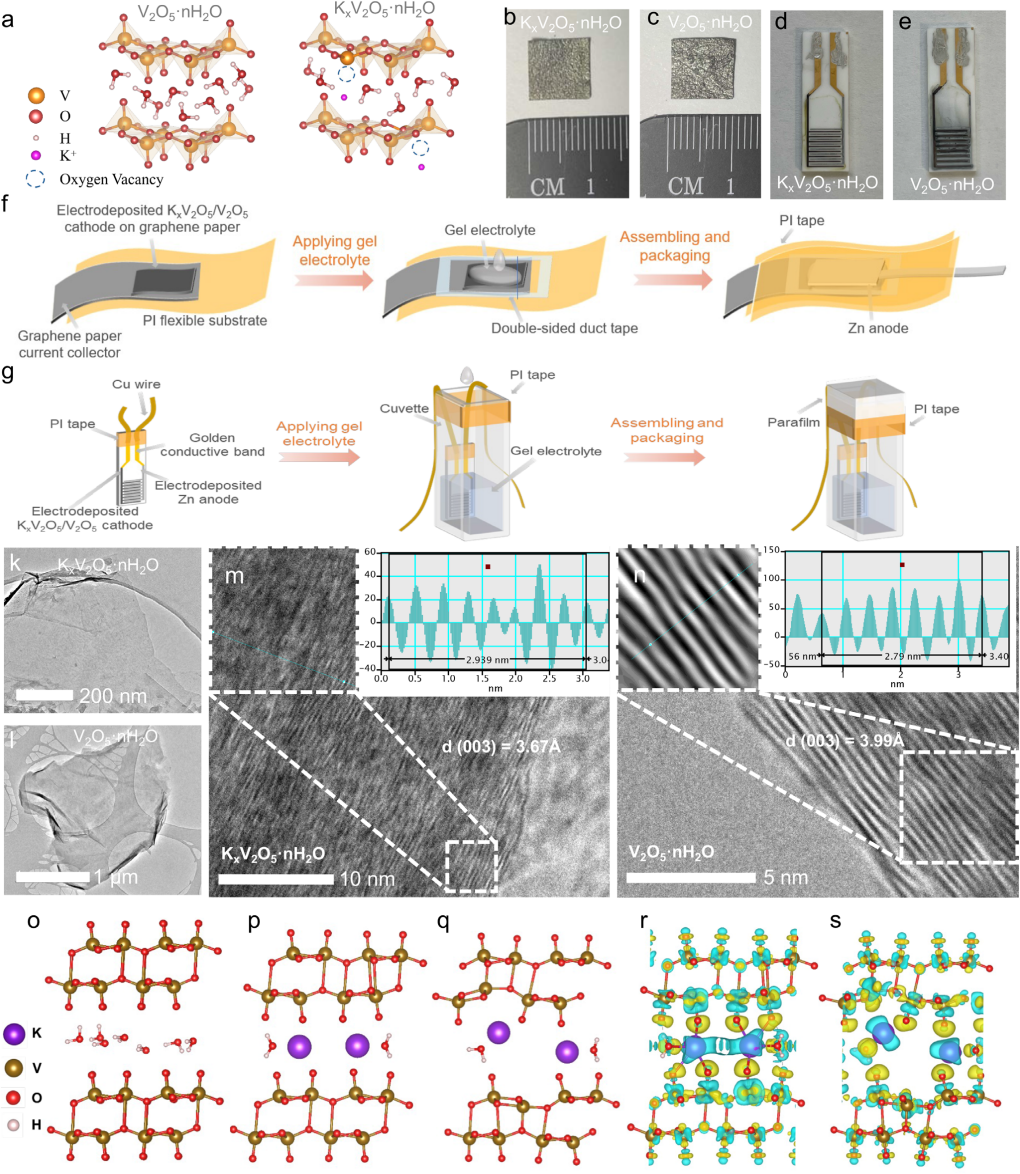
(a) Schematic illustration of the structure for V_2_O_5_·nH_2_O (left) and K_x_V_2_O_5_·nH_2_O (right). Digital pictures of (b) K_x_V_2_O_5_·nH_2_O, (c) V_2_O_5_·nH_2_O on graphene paper, as well as (d) K_x_V_2_O_5_·nH_2_O//Zn, (e) V_2_O_5_·nH_2_O//Zn MB chips, and their corresponding schematic representation of the fabrication process for (f) Zn‐TFBs as well as for (g) Zn‐MBs. TEM (k, l) and (m, n) HRTEM images of K_x_V_2_O_5_·nH_2_O and V_2_O_5_·nH_2_O with inverse fast Fourier transform (FFT) graph, intensity profile, and calculated d‐spacing for respective (003) crystal planes. Optimized structures of (o) V_2_O_5_·nH_2_O with *n* = 0.44, (p) K_x_V_2_O_5_·nH_2_O, and (q) K_x_V_2_O_5__−__x_·nH_2_O where *x* = 0.25 and *n* = 0.25. The golden, red, off‐white, and violet spheres represent vanadium, oxygen, hydrogen, and potassium atoms, respectively. Charge density difference (CDD) plot for (r) K_x_V_2_O_5_·nH_2_O and (s) K_x_V_2_O_5__−__x_·nH_2_O. The yellow color represents the charge accumulation region, while the cyan color indicates the charge depletion region, with an iso‐surface value of 0.002 e Å^−^^3^.

The cathode materials were fabricated via an electrodeposition technique, enabling direct growth on current collectors without the need for binders or conductive additives. Precursor solutions containing VOSO_4_ and K_2_SO_4_ were used for deposition in a three‐electrode system (see Experimental Section for details). Through this process, two types of materials were obtained: V_2_O_5_·nH_2_O, in which only structural water molecules are intercalated between V_2_O_5_ layers, and K_x_V_2_O_5_·nH_2_O, where both structural water molecules and K^+^ ions are co‐intercalated, as schematically illustrated in Figure 1a. For electrochemical testing, the electrodeposited materials on graphene paper (Figure 1b,c) were employed as cathodes in coin cells and Zn‐TFBs, while depositions on Au interdigitated electrodes (IDEs) (Figure 1d,e) served as cathodes for Zn‐MBs. Assembly details for coin cells, Zn‐TFBs, and Zn‐MBs are provided in the Supporting Information. The coin cells and Zn‐TFBs were tested against commercial Zn foil anodes (Figure 1f), whereas in Zn‐MBs, Zn anodes were electrodeposited to form K_x_V_2_O_5_·nH_2_O//Zn and V_2_O_5_·nH_2_O//Zn MBs (Figure 1g). The morphologies of K_x_V_2_O_5_·nH_2_O and V_2_O_5_·nH_2_O were examined by scanning electron microscopy (SEM). Low‐ and high‐magnification images of the electrodeposited films on graphene paper (Figure S1a–f) revealed successful and uniform deposition of both materials, displaying surface textures consistent with the underlying substrate. Cross‐sectional SEM images (Figure S1g–i) showed that the interlamellar spacing of the multilayer graphene substrate expanded after deposition of the active materials, leading to a significant increase in specific surface area. Transmission electron microscopy (TEM) further confirmed the flake‐like nanostructure of both active materials (Figure 1k,l). High‐resolution TEM (HRTEM) images (Figure 1m,n) revealed lattice spacings corresponding to the (003) crystal planes, measured as 3.67 Å for K_x_V_2_O_5_·nH_2_O and 3.99 Å for V_2_O_5_·nH_2_O. The reduced d‐spacing indicates that K^+^ pre‐intercalation induces a slight contraction of the interlayer distance. Energy‐dispersive spectroscopy (EDS) elemental mapping (Figure S2) confirmed the presence and uniform distribution of O (Figure S2b), V (Figure S2c), and K (Figure S2d) within the K_x_V_2_O_5_·nH_2_O nanoflakes, while V_2_O_5_·nH_2_O contained only O (Figure S2f) and V (Figure S2g).

To elucidate the reduced d‐spacing observed in K_x_V_2_O_5_·nH_2_O compared to V_2_O_5_·nH_2_O, and to account for the Coulombic attraction between intercalated K^+^ ions and electronegative oxygen atoms, we further performed first‐principles calculations based on Density Functional Theory (DFT). We considered an orthorhombic crystal system within the space group Pmmn, consistent with experimental observations. Detailed structural information on the double‐layered slab and bilayer sheet of orthorhombic V_2_O_5_ is provided in the Supporting Information. Previous reports on hydrated V_2_O_5_·nH_2_O describe the incorporation of H_2_O molecules between stacked V–O layers of α‐V_2_O_5_, leading to structural deformation that has been modeled using a monoclinic crystal system. In our approach, we adopted key structural parameters from these reported monoclinic models, such as interlayer spacing, the separation between adjacent layers within a bilayer sheet, and incorporated them into our orthorhombic Pmmn framework. The intercalated water molecules play a vital role in stabilizing this layered framework. Importantly, the interlayer spacing demonstrates a high sensitivity to both the level of hydration (as indicated by the value of n in V_2_O_5_·nH_2_O) and the presence of intercalated ions, aspects that are thoroughly examined in the context of this study. The optimized structure of V_2_O_5_·nH_2_O, with n equal to 0.44, is presented in Figure 1o and exhibits an interlayer distance of 12.11 Å, which aligns well with experimental observations (see further). To explore the effect of alkali ion intercalation, potassium atoms (K) were introduced between the bilayers as shown in Figure 1p,q. Interestingly, despite the relatively large ionic radius of K^+^, the interlayer distance in K_x_V_2_O_5_·nH_2_O (where *x* = 0.25 and *n* = 0.25 which is close to the water content detected further) decreased to 11.01 Å. Additionally, in the case of oxygen‐deficient potassium‐intercalated vanadium oxide, specifically K_x_V_2_O_5__−__x_·nH_2_O (with *x* = 0.25 and *n* = 0.25), the interlayer spacing slightly increased to 11.23 Å. Since the water content is assumed to be the same, this counterintuitive reduction in interlayer spacing is attributed to Coulombic attractive interactions between the intercalated K^+^ ions and the electronegative oxygen atoms in the vanadium oxide layers. This phenomenon is validated through charge density difference (CDD) analysis, which is derived from the following equation:

$$\Delta \rho =\rho_{K_{x}V_{2}O_{5}\cdot nH_{2}O}-\rho_{V_{2}O_{5}\cdot nH_{2}O}-\rho_{K_{x}}$$

In this equation, the charge density of the K intercalated V_2_O_5_·nH_2_O layer is represented by $\rho_{K_{x}V_{2}O_{5}\cdot nH_{2}O}$, and $\rho_{V_{2}O_{5}\cdot nH_{2}O}$ represents the charge density of the V_2_O_5_·nH_2_O layer, while $\rho_{K_{x}}$ denotes the charge density of the isolated K atoms. As illustrated in Figure 1r,s, the charge density difference (CDD) plot demonstrates charge depletion (represented by cyan regions) surrounding the potassium (K) atoms, in addition to charge accumulation (indicated by yellow regions) near the oxygen (O) atoms in the host lattice. The observed loss of electron density adjacent to the K atom implies its oxidation to K^+^, while the accumulation of charge near the oxygen atoms signifies a notable transfer of charge from K to the V_2_O_5_·nH_2_O host material. This electrostatic interaction between K^+^ ions and the negatively charged oxygen atoms can result in a decrease in interlayer spacing, as the attractive forces exert a pulling effect that draws the layers closer together.

**FIGURE 2:**
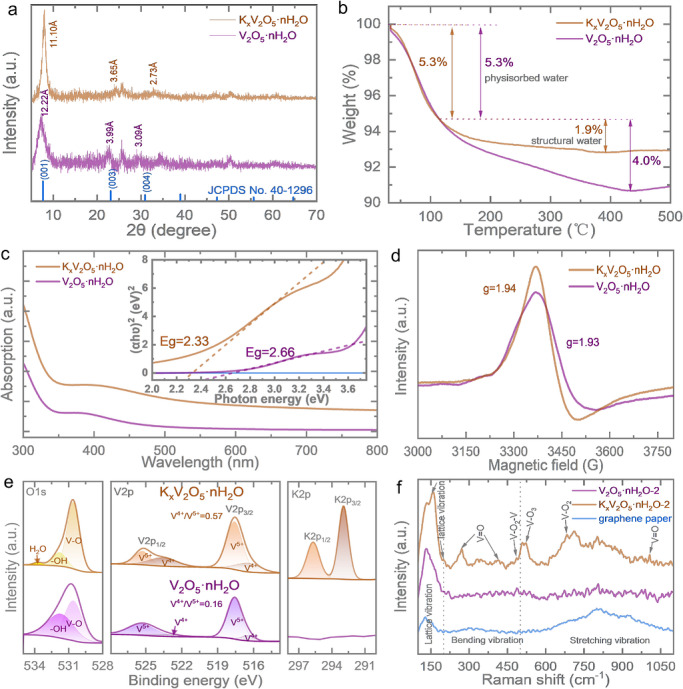
(a) XRD patterns, (b) TGA curves, (c) UV–vis absorption spectra, (d) EPR results, (e) O1s, V2p, K2p high‐resolution XPS spectra, and (f) Raman spectra of K_x_V_2_O_5_·nH_2_O and V_2_O_5_·nH_2_O.

Figure 2a presents the X‐ray diffraction (XRD) patterns of V_2_O_5_·nH_2_O and K_x_V_2_O_5_·nH_2_O. The crystal symmetry of V_2_O_5_·nH_2_O matches JCPDS No. 40–1296, confirming its orthorhombic symmetry with the Pmmm space group, and the XRD pattern is consistent with previously reported structures of V_2_O_5_·nH_2_O [31, 32, 33]. The first Bragg reflection at 2θ = 6.7° corresponds to the (001) lattice plane, exhibiting the highest peak intensity and indicating a strong *c*‐axis preferred orientation of the V_2_O_5_·nH_2_O structure, which arises from the regular stacking of these layers. Therefore, this reflection directly represents the interlayer spacing between adjacent V_2_O_5_ sheets [34, 35]. The spacing d_001_ can be determined using Bragg's law: $d_{001}=\frac{\lambda }{2sin\theta }$, where λ is the wavelength of the XRD radiation, d is the interlayer spacing, and θ is the diffraction angle, which is calculated to be 12.22 Å for V_2_O_5_·nH_2_O [36]. The position of the (001) peak reflects the amount of interlayer water present and therefore changes during hydration or dehydration processes, which cause expansion or contraction of the layered structure [36, 37]. The XRD pattern of K_x_V_2_O_5_·nH_2_O is very similar to that of V_2_O_5_·nH_2_O, indicating that the c‐axis preference and layered structure remain unaffected by K^+^ intercalation. However, the (001) peak shifts to a higher 2θ value (7.96°), corresponding to a reduced d_001_ lattice constant of 11.10 Å. Additionally, the calculated lattice spacings for K_x_V_2_O_5_·nH_2_O are d_003_ = 3.65Å, d_004_ = 2.73Å, compared to d_003_ = 3.99Å, d_004_ = 3.09Å for V_2_O_5_·nH_2_O. The intercalation of K^+^ into V_2_O_5_·nH_2_O causes the (001), (003), and (004) diffraction peaks to shift toward higher 2θ values, confirming a reduction in interlayer spacing, consistent with the first‐principles calculations discussed earlier. Figure 2b presents the thermogravimetric analysis (TGA) curves of both powder samples, revealing two distinct stages of weight loss. The initial drop from 100% to 94.7% is primarily attributed to the evaporation of physisorbed water, which mainly comes from ambient air moisture [21]. The second stage corresponds to the release of structural water, with weight losses of 1.9% for K_x_V_2_O_5_·nH_2_O and 4.0% for V_2_O_5_·nH_2_O, giving estimated molar ratios (n) of ∼ 0.20–0.21 and 0.42, respectively [21]. These results indicate that K^+^ insertion reduces the amount of intercalated water in the structure. On the other hand, a decrease in the optical bandgap is observed in K_x_V_2_O_5_·nH_2_O compared to V_2_O_5_·nH_2_O (Figure 2c). The absorption peak edge for K_x_V_2_O_5_·nH_2_O appears at ∼ 419 nm, redshifted relative to ∼392 nm for V_2_O_5_·nH_2_O. This shift corresponds to a reduction in bandgap energy from 2.66 to 2.33 eV, as determined from the Tauc plot. The decrease in bandgap enhances the electronic conductivity of the material [38, 39]. This improvement arises from the generation of oxygen vacancies, which introduce defect states between the conduction and valence bands, allowing electrons to transition more readily into the conduction band. Such enhanced conductivity is particularly critical when cathode materials are employed in TFBs and MBs, where conductive additives are typically absent [40]. The presence of oxygen vacancies is confirmed by electron paramagnetic resonance (EPR) measurements (Figure 2d), which reveal signals corresponding to paramagnetic V^4^^+^ (d^1^) centers. The signal is due to aggregated V^4+^ ions, and the as observed broad asymmetric single line can be due to the relaxation processes effecting the surface V^4+^ paramagnetic centers (PCs) [41]. With the K^+^ intercalation, partial reduction of V^5+^ to V^4+^ increases the concentration of EPR active species (see further), as observed from the enhanced signal intensity in the K_x_V_2_O_5_·nH_2_O sample [42]. The slight line narrowing suggests a more uniform electronic environment, likely resulting from charge compensation by K^+^ ions. Its g‐value shift from 1.93 to 1.94, can be attributed to increased spin–orbit coupling and local asymmetry around V^4+^ centers induced by K^+^ insertion. Oxygen vacancies form due to the valence imbalance among vanadium ions and play an important role in weakening Zn^2+^‐V‐O interactions, thereby facilitating faster Zn^2+^ diffusion to the lattice (see further) [43]. The existence of elements O, V in V_2_O_5_·nH_2_O cathode and O, V, K in K_x_V_2_O_5_·nH_2_O cathode can be confirmed again by X‐ray photoelectron spectroscopy (XPS) surveys in Figure S3a,b. O1s, V2p, K2p high‐resolution XPS spectra for two active materials can be depicted from Figure 2e. O1s for K_x_V_2_O_5_·nH_2_O can be split into three peaks at binding energies of ∼ 533.7, ∼ 531.8, and ∼ 530.6 eV belonging to O signals from physiosorbed water, structural water, V_2_O_5_ lattice V–O [30]. Two V2p curves both consist of V2p_1/2_ and V2p_3/2_ peaks. To determine V element valence state, they can be deconvoluted into V^5+^2p_1/2_, V^4+^2p_1/2_, V^5+^2p_3/2_, V^4+^2p_3/2_ at ∼ 525.3, ∼ 523.6, ∼ 517.6, and ∼ 516.4 eV for K_x_V_2_O_5_·nH_2_O, at ∼ 525.4, ∼ 523.7, ∼ 517.6, and ∼ 516.5 eV for V_2_O_5_·nH_2_O. The mixed valence system, result from oxygen defects, in K_x_V_2_O_5_·nH_2_O has a higher V^4+^/V^5+^ ratio of 0.57 than 0.16 in V_2_O_5_·nH_2_O, which endow K_x_V_2_O_5_·nH_2_O cathode with improved electronic conductivity and contribute to electrochemical capacity eventually (see further) [27]. Comparing with V_2_O_5_·nH_2_O, two additional peaks K2p_1/2_ and K2p_3/2_ for K_x_V_2_O_5_·nH_2_O can be observed at ∼ 295.7 and ∼ 293.0 eV, which proves the pre‐insertion of K^+^ between layers of V_2_O_5_·nH_2_O [44]. The Raman characteristic peaks for K_x_V_2_O_5_·nH_2_O cathode, V_2_O_5_·nH_2_O cathode and graphene paper can be observed in Figure 2f. Except the peak from graphene paper substrate around 130 cm^−1^, most peaks for V_2_O_5_·nH_2_O are too weak to be detected, which comes from a higher water content between layered structure [45]. In K_x_V_2_O_5_·nH_2_O, more peaks can be discovered. The lattice vibration is assigned to ∼ 156 and ∼ 197 cm^−1^, the later one is subjected to compressive deformations along the a direction [46]. Peaks for V═O are locate at ∼ 271, ∼ 412 and ∼ 1006 cm^−1^, the former two are attributed to bending vibration, the last one is stretching mode. Bridging V–O_2–_V bending vibration appear separately at ∼ 484 cm^−1^. The peaks at ∼ 522, ∼ 683 cm^−1^ are aligned with stretching mode for triply coordinated edge‐shared oxygen (V–O_3_) and doubly coordinated corner‐shared oxygen (V–O_2_) [46].

To evaluate the charge storage performance of electrodeposited thin‐film V_2_O_5_·nH_2_O and K_x_V_2_O_5_·nH_2_O, the as‐prepared samples were assembled as cathodes in zinc‐ion coin cells using 3 m Zn(CF_3_SO_3_)_2_ electrolyte. Cyclic voltammetry (CV) curves of K_x_V_2_O_5_·nH_2_O‐2 (2 h electrodeposition), K_x_V_2_O_5_·nH_2_O‐4 (4 h electrodeposition), V_2_O_5_·nH_2_O‐2, and V_2_O_5_·nH_2_O‐4 coin cells at scan rates of 0.2 and 1.0 mV s^−^^1^ are compared in Figure S4a,b. Within the potential window of 0.2–1.6 V, two pairs of redox peaks are observed in each CV curve, corresponding to Zn^2^^+^ intercalation/deintercalation in the cathode. Peaks 1 and 2 are associated with the V^5^^+^/V^4^^+^ redox couple, while peaks 3 and 4 correspond to the V^4^^+^/V^3^^+^ transition. At identical electrodeposition times, the areal current of K_x_V_2_O_5_·nH_2_O is significantly higher than that of V_2_O_5_·nH_2_O, indicating enhanced capacity. As the electrodeposition time doubled, the areal currents are increased in both K_x_V_2_O_5_·nH_2_O‐4 and V_2_O_5_·nH_2_O‐4 coin cells due to the higher mass loading. To understand the charge storage mechanism in four coin cells, we performed some *b* values calculated from Figure S4c–f at a scan rate range of 0.2–1.0 mV/s. The *b* values can be calculated via the relationship formula between peak current of redox peaks (*i_p_*) and scan rates (*v*): *i_p_* =  *av^b^*, in which *a* and *b* are both material‐dependent parameters. When *b* is closer to 0.5, the charge storage process in the cathode material is diffusion‐control dominated. When *b* is closer to 1.0, the charge storage process is dominantly capacitive‐controlled. The linear‐fitted *b* values for all cells, which are computed in Figure S5a–d and summarized in Figure S5e, are close to 1.0 at their respective redox peaks, indicating capacitive‐dominated charge storage behavior. The comparative galvanostatic discharge‐charge (GDC) profiles of K_x_V_2_O_5_·nH_2_O‐2, K_x_V_2_O_5_·nH_2_O‐4, V_2_O_5_·nH_2_O‐2, and V_2_O_5_·nH_2_O‐4 electrodes tested in coin cells at 50 and 1000 µA cm^−^^2^ are shown in Figure S6a,b. The K_x_V_2_O_5_·nH_2_O‐2 cell delivers areal capacities of 367.9 and 333.8 µAh cm^−^^2^ at 50 and 1000 µA cm^−^^2^, respectively—values that are nearly double those of V_2_O_5_·nH_2_O‐2 (181.6 and 152.7 µAh cm^−^^2^). As summarized in Figure S6c–f, K_x_V_2_O_5_·nH_2_O‐2 consistently achieves more than twice the capacity of V_2_O_5_·nH_2_O‐2 across the full areal current range (50–1000 µA cm^−^^2^), while K_x_V_2_O_5_·nH_2_O‐4 exhibits at least 1.8 × higher capacity than V_2_O_5_·nH_2_O‐4. The enhanced performance of K^+^‐intercalated electrodes arises from multiple synergistic factors: the generation of oxygen vacancies that create additional H^+^ and Zn^2^^+^ diffusion channels [22], extra active sites that facilitate redox reactions [28], and a mixed‐valence V^4^^+^/V^5^^+^ system that improves electronic conductivity [27]. Electrochemical impedance spectroscopy (EIS) further supports this conclusion: as shown in Figure S7, K_x_V_2_O_5_·nH_2_O electrodes exhibit lower charge‐transfer resistance (*R_ct_*) than V_2_O_5_·nH_2_O at identical deposition times, indicating reduced interfacial impedance in the K^+^‐intercalated cathodes. Additionally, Figure S6a–f reveal that electrodes with longer deposition times (K_x_V_2_O_5_·nH_2_O‐4 and V_2_O_5_·nH_2_O‐4) possess higher areal capacities than their 2 h counterparts, primarily due to increased mass loading. Rate capability tests (Figure S8a) further confirm this trend. For instance, K_x_V_2_O_5_·nH_2_O‐2 delivers areal capacities of ∼368, 364, 360, 349, and 334 µAh cm^−^^2^ at areal currents of 50, 100, 200, 500, and 1000 µA cm^−^^2^, respectively. In contrast, V_2_O_5_·nH_2_O‐2 exhibits lower areal capacities of 182, 178, 172, 162, and 153 µAh cm^−^^2^ at the same areal currents. Upon returning the areal current to 50 µA cm^−^^2^, both electrodes nearly recover their original capacity, demonstrating good reversibility. Similar rate tests on K_x_V_2_O_5_·nH_2_O‐4 and V_2_O_5_·nH_2_O‐4 further confirm that prolonged electrodeposition enhances mass loading, thereby delivering significantly higher capacities while maintaining good rate retention. The cycling performance of the four coin cells at an areal current of 1000 µA cm^−^^2^ is shown in Figure S8b. Interestingly, the capacities gradually increase with cycling, likely due to the slow activation of the materials at high current, a phenomenon commonly observed in V_2_O_5_‐based cathodes for Zn‐ion batteries [47]. After 5000 cycles, K_x_V_2_O_5_·nH_2_O‐2 retains 100% of its initial areal capacity, far surpassing the 55% retention of V_2_O_5_·nH_2_O‐2. This striking difference highlights the advantages of K^+^ incorporation. It is expected that K^+^ acts as a structural pillar, preventing collapse of the layered framework [23]. Moreover, K^+^‐induced oxygen vacancies provide additional free volume, alleviating the stress associated with protons (H^+^) and Zn^2^^+^ insertion/extraction (see further) [26]. For K_x_V_2_O_5_·nH_2_O‐4 and V_2_O_5_·nH_2_O‐4 coin cells, the continuous charge–discharge cycling is sustained for only ∼260 and ∼160 cycles, respectively. The poorer reversibility at longer electrodeposition times can be attributed to higher material loading, which increases the risk of delamination from the current collector in the absence of binders and conductive additives, ultimately accelerating capacity fading. Experimentally, these effects are difficult to isolate in coin cells due to additional degradation of glass fiber separators, which interact with both electrodes during extended cycling. Hence, compared with pristine V_2_O_5_·nH_2_O, K^+^ pre‐intercalation endows K_x_V_2_O_5_·nH_2_O with superior capacity and cycling stability. However, although longer electrodeposition improves areal capacity, it inevitably compromises long‐term durability. Therefore, K_x_V_2_O_5_·nH_2_O‐2 and V_2_O_5_·nH_2_O‐2, which balance capacity and stability, will be the focus of further investigations in this work.

In aqueous ZIBs employing mildly acidic electrolytes such as 3 m Zn(CF_3_SO_3_)_2_, charge compensation is not exclusively governed by Zn^2^^+^ ions; H^+^ also actively participates in the electrochemical process. The hydrated H^+^ possesses an extremely small effective size (∼0.89 × 10^−^^6^ nm), several orders of magnitude lower than that of Zn^2^^+^, and displays a substantially higher diffusion coefficient in water (9.3 × 10^−^^5^ cm^2^ s^−^^1^) compared with Zn^2^^+^ (0.7 × 10^−^^5^ cm^2^ s^−^^1^) [48, 49]. These characteristics render H^+^ a kinetically favorable carrier in cathode materials during charge storage. Furthermore, the presence of oxygen vacancies in layered oxide frameworks such as V_2_O_5_ induces local band‐structure perturbations and weakens ion‐lattice interactions, generating energetically accessible hopping sites that facilitate rapid proton migration. When structural water is retained within the interlayer spacing, H^+^ transport is further accelerated through hydrogen‐bonded chains via the Grotthuss hopping mechanism, enabling efficient long‐range proton conduction. Enhancing the contribution of H^+^ therefore directly promotes Faradaic kinetics and improves high‐rate electrochemical performance. As discussed previously, K^+^ pre‐intercalation markedly increases the oxygen‐vacancy density in K_x_V_2_O_5_·nH_2_O relative to pristine V_2_O_5_·nH_2_O. To quantitatively assess the role of proton‐only charge storage, cathodes were tested in a Zn^2^^+^‐free aqueous electrolyte prepared by adjusting deionized water with H_2_SO_4_ to a pH of 3.98, which equals to the pH of aqueous 3 m Zn(CF_3_SO_3_)_2_. Under these conditions (Figure S9a), the K_x_V_2_O_5_·nH_2_O electrode delivered more than twice the capacity of V_2_O_5_·nH_2_O, while commercial V_2_O_5_ with negligible vacancy content exhibited only minimal H^+^‐related storage. These observations confirm that both V_2_O_5_·nH_2_O and K_x_V_2_O_5_·nH_2_O contain oxygen defects (Figure 2d), and that K^+^ incorporation further amplifies vacancy formation, thereby substantially strengthening the proton‐storage contribution. To further elucidate the role of oxygen vacancies in proton storage, hydrogen adsorption on V_2_O_5_ substrates was investigated by DFT using: $E_{b}=E_{H@\mathrm{substrate}}\;-E_{\mathrm{substrate}}-\frac{1}{2}E_{H_{2}}$; where E_H@substrate_, E_substrate_, and $E_{H_{2}}$ are the total energies of the H adsorbed substrate, bare substrate and the hydrogen molecule, respectively. As shown in Figure S9b, oxygen‐deficient V_2_O_5_ exhibits a strong affinity toward hydrogen with a binding energy of −2.72 eV, higher than that of pristine V_2_O_5_ (−2.65 eV). This result highlights the critical role of oxygen vacancies in facilitating proton transfer within the vanadium oxide lattice. The absence of oxygen atoms generates under‐coordinated neighboring oxygen sites that preferentially attract and stabilize protons. Meanwhile, vacancy‐induced lattice distortions modify O–O distances, promoting proton mobility via a Grotthuss‐type hopping mechanism. Furthermore, the removal of O^2^^−^ anions redistributes local electron density around adjacent metal centers, altering the electrostatic environment and stabilizing transient proton configurations. Collectively, these effects lower the energy barriers for proton migration and intercalation/deintercalation, significantly enhancing proton mobility and overall ion‐transport kinetics. These findings confirm that charge storage in the studied V_2_O_5_ cathodes is not governed solely by Zn^2^^+^ intercalation; rather, H^+^ participation plays an equally crucial role in boosting the overall capacity and rate performance.

To evaluate their compatibility with thin‐film configurations, the electrochemical behavior of K_x_V_2_O_5_·nH_2_O‐2 and V_2_O_5_·nH_2_O‐2 Zn‐TFBs was investigated using a guar‐gum (GG) gel electrolyte. The CV curves of V_2_O_5_·nH_2_O‐2 Zn‐TFBs collected in the voltage window of 0.2–1.6 V at scan rates ranging from 0.2 to 1.0 mV s^−^^1^ are presented in Figure S10b. In general, aqueous electrolytes produce more pronounced redox features than gel matrices due to the higher activation energy (*E_a_*) and increased ion‐transport resistance in polymeric gels [50]. Consequently, the V_2_O_5_·nH_2_O‐2 coin cell operated in aqueous electrolyte exhibits two well‐defined pairs of redox peaks, whereas the Zn‐TFB employing the GG gel electrolyte shows only a single dominant redox couple at 0.88/1.10 V (0.2 mV s^−^^1^), corresponding to the V^4^^+^/V^5^^+^ redox transition. In contrast, the K_x_V_2_O_5_·nH_2_O‐2 Zn‐TFBs retain two pairs of prominent redox peaks (Figure S10a) even in the gel electrolyte, closely resembling their behavior in aqueous coin‐cell configurations. This observation further confirms that K_x_V_2_O_5_·nH_2_O‐2 cathodes are well suited for thin‐film batteries using gel electrolyte matrices. To validate the higher *E_a_* of the GG gel electrolyte, EIS was conducted on Zn//Zn symmetric cells with 3 m Zn(CF_3_SO_3_)_2_ aqueous and GG gel electrolytes. Nyquist plots measured from 25°C–65°C (Figure S11a,b) were fitted using the Arrhenius equation, with the extracted *E_a_* values summarized in Figure S11c. The *E_a_* of the gel electrolyte (38.6 kJ mol^−^^1^) is higher than that of the aqueous electrolyte (34.6 kJ mol^−^^1^), indicating that Zn^2^^+^ desolvation and migration at the gel electrolyte/electrode interface require more energy [50]. The ionic conductivity of the GG electrolyte was further determined using EIS on SS//SS symmetric cells (Figure S12). Calculated values are summarized in Tables S1 and S2, showing consistently lower ionic conductivity for the GG electrolyte compared to the aqueous electrolyte across all temperatures, confirming slower ion mobility and greater diffusion resistance. To further understand this limitation, viscosity measurements were performed (Figure S13), revealing viscosities of 1.26 P for the gel electrolyte and 0.6 P for the aqueous electrolyte at 50°C. The higher viscosity of the gel electrolyte increases Zn^2^^+^ diffusion resistance, thereby reducing ionic conductivity.

**FIGURE 3:**
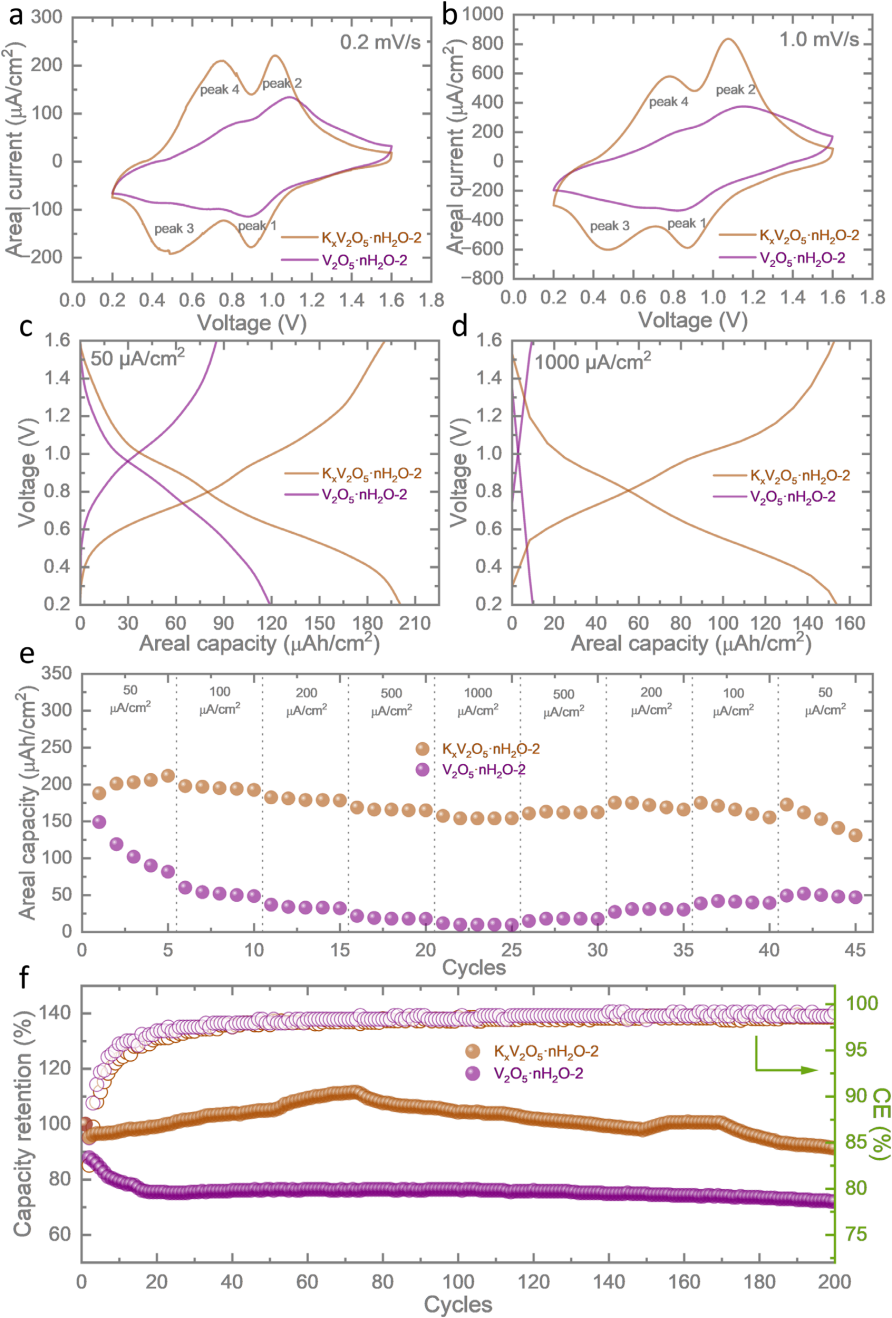
Comparative CV curves of K_x_V_2_O_5_·nH_2_O‐2 and V_2_O_5_·nH_2_O‐2 Zn‐TFBs with GG gel electrolyte at (a) 0.2 mV s^−^^1^ and (b) 1.0 mV s^−^^1^. Comparative GDC profiles at areal currents of (c) 50 µA cm^−^^2^ and (d) 1000 µA cm^−^^2^. (e) Rate capability from 50 to 1000 µA cm^−^^2^ and (f) cycling performance at 1000 µA cm^−^^2^.

The comparative CV curves of K_x_V_2_O_5_·nH_2_O‐2 and V_2_O_5_·nH_2_O‐2 Zn‐TFBs at 0.2 and 1.0 mV s^−^^1^ are shown in Figure 3a,b. Unlike V_2_O_5_·nH_2_O‐2, the K_x_V_2_O_5_·nH_2_O‐2 electrode exhibits two distinct pairs of redox peaks and significantly higher areal currents. This pre‐intercalation increases the V^4^^+^/V^5^^+^ ratio, providing more V^4^^+^ for the V^3^^+^/V^4^^+^ redox reaction, facilitating efficient electron transfer. Additionally, K^+^ incorporation enhances electronic conductivity, while the introduction of oxygen vacancies enables cooperative H^+^/Zn^2^^+^ co‐storage and markedly accelerated reaction kinetics to mitigate the increased diffusion resistance and activation energy (*E_a_*) associated with the gel electrolyte, particularly for the V^3^^+^/V^4^^+^ reaction [7, 22, 27, 28]. Consequently, both redox couples proceed more vigorously, resulting in improved capacity. At 0.2 mV s^−^^1^, K_x_V_2_O_5_·nH_2_O‐2 shows peaks at 0.90/1.02 V (V^4^^+^/V^5^^+^) and 0.49/0.76 V (V^3^^+^/V^4^^+^). The narrower potential gap between the anodic and cathodic peaks of the V^4^^+^/V^5^^+^ couple in K_x_V_2_O_5_·nH_2_O‐2 compared to V_2_O_5_·nH_2_O‐2 indicates improved reversibility. Figure S14a,b presents the *b*‐values derived from peak currents. For K_x_V_2_O_5_·nH_2_O‐2, the *b*‐value of 0.83 at peak 2 suggests capacitance‐dominated charge storage for the V^4^^+^→V^5^^+^ transition, while values of 0.74, 0.71, and 0.64 at other peaks indicate diffusion‐controlled processes. In contrast, V_2_O_5_·nH_2_O‐2 exhibits lower *b*‐values of 0.67 and 0.63, implying a stronger reliance on diffusion‐controlled storage. Further insight into the charge storage mechanism was obtained by deconvoluting the capacitive (*k*_1_ *v*) and diffusion‐controlled (k_2_v^0.5^) contributions from the current—scan rate relationship, i(*v*)  =  k_1_v + k_2_v^0.5^, reformulated as $i\left(v\right)/v^{0.5}=k_{1}v^{0.5}+k_{2}$ [51]. The results (Figure S14c) reveal that in K_x_V_2_O_5_·nH_2_O‐2, diffusion‐controlled contributions dominate at lower scan rates (60% → 52% from 0.2 to 0.5 mV s^−^^1^). However, as the scan rate increases above 0.6 mV s^−^^1^, the diffusion contribution decreases (49% → 40%), while capacitive behavior becomes more prominent. In comparison, V_2_O_5_·nH_2_O‐2 Zn‐TFBs remain diffusion‐dominated across the entire scan rate range, with diffusion contributions decreasing from 75% at 0.2 mV s^−^^1^ to 58% at 1.0 mV s^−^^1^. The capacitive contributions of both systems at 0.2 and 1.0 mV s^−^^1^ are highlighted by the shaded areas in the CV curves (Figure S10c–f). The noticeably larger capacitive current contribution in K_x_V_2_O_5_·nH_2_O‐2 compared to V_2_O_5_·nH_2_O‐2 further supports its cooperative H^+^/Zn^2^^+^ co‐storage and markedly accelerated reaction kinetics.

The areal capacities of K_x_V_2_O_5_·nH_2_O‐2 and V_2_O_5_·nH_2_O‐2 Zn‐TFBs at 50 and 1000 µA cm^−^^2^ are shown in Figures 3c,d. Benefiting from K^+^ pre‐insertion, K_x_V_2_O_5_·nH_2_O‐2 delivers 200.9 µAh cm^−^^2^ at 50 µA cm^−^^2^ and 154.0 µAh cm^−^^2^ at 1000 µA cm^−^^2^, far surpassing the corresponding values of V_2_O_5_·nH_2_O‐2 (119.0 and 9.8 µAh cm^−^^2^). As the areal current increases from 50 to 1000 µA cm^−^^2^, K_x_V_2_O_5_·nH_2_O‐2 maintains high areal capacities from 200.9 to 154.0 µAh cm^−^^2^ (Figure S15a), whereas V_2_O_5_·nH_2_O‐2 rapidly declines from 119.0 to 9.8 µAh cm^−^^2^ (Figure S15b). Rate tests (Figure 3e) clearly highlight the superior rate capability of K_x_V_2_O_5_·nH_2_O‐2. At high areal currents, the limited diffusion channels and active sites in V_2_O_5_·nH_2_O restrict cooperative H^+^/Zn^2^^+^ intercalation/extraction, leading to drastic capacity fading. By contrast, K_x_V_2_O_5_·nH_2_O‐2 benefits from more oxygen vacancies that provide additional diffusion channels and active sites, while K^+^ incorporation reduces electron mobility barriers, unlock a highly efficient proton‐Zn^2^^+^ co‐storage mechanism, and enables rapid ion transport. EIS further supports these results. The Nyquist plots (Figure S16a) show a smaller charge‐transfer resistance (*R_ct_*) for K_x_V_2_O_5_·nH_2_O‐2 compared to V_2_O_5_·nH_2_O‐2. Correspondingly, the Warburg coefficients (σ) fitted from the low‐frequency region (Figure S16b) are 38.4 for K_x_V_2_O_5_·nH_2_O‐2 and 127.5 for V_2_O_5_·nH_2_O‐2, indicating a much higher Zn^2^^+^ diffusion coefficient in the K^+^‐intercalated material. Long‐term cycling tests (Figure 3f) confirm the stability of the K_x_V_2_O_5_·nH_2_O‐2 framework. After 200 cycles at 1000 µA cm^−^^2^, it retains 91% of its initial capacity, while V_2_O_5_·nH_2_O‐2 loses 28%. Self‐discharge behavior was also assessed (Figure S17). After charging to 1.6 V at 1000 µA cm^−^^2^, V_2_O_5_·nH_2_O‐2 retains 88% of its maximum voltage (1.4 V), whereas K_x_V_2_O_5_·nH_2_O‐2 retains 93% (1.5 V) for more than 190 h—superior to many previously reported planar energy storage devices. For example, an AC‐based symmetric micro‐supercapacitor (SMS) retained 50% voltage after 2.5 h [52], printed Na_x_MnO_2_ SMS after 7 h [53], inkjet‐printed Fe‐MnO_2_ SMS after 3 h [54], AC SMS with isocyanate‐based additive after 30 h [55], laser‐written GO film SMS after 13 h [56], and laser‐written rGO SMS in less than 1 h [57].

**FIGURE 4:**
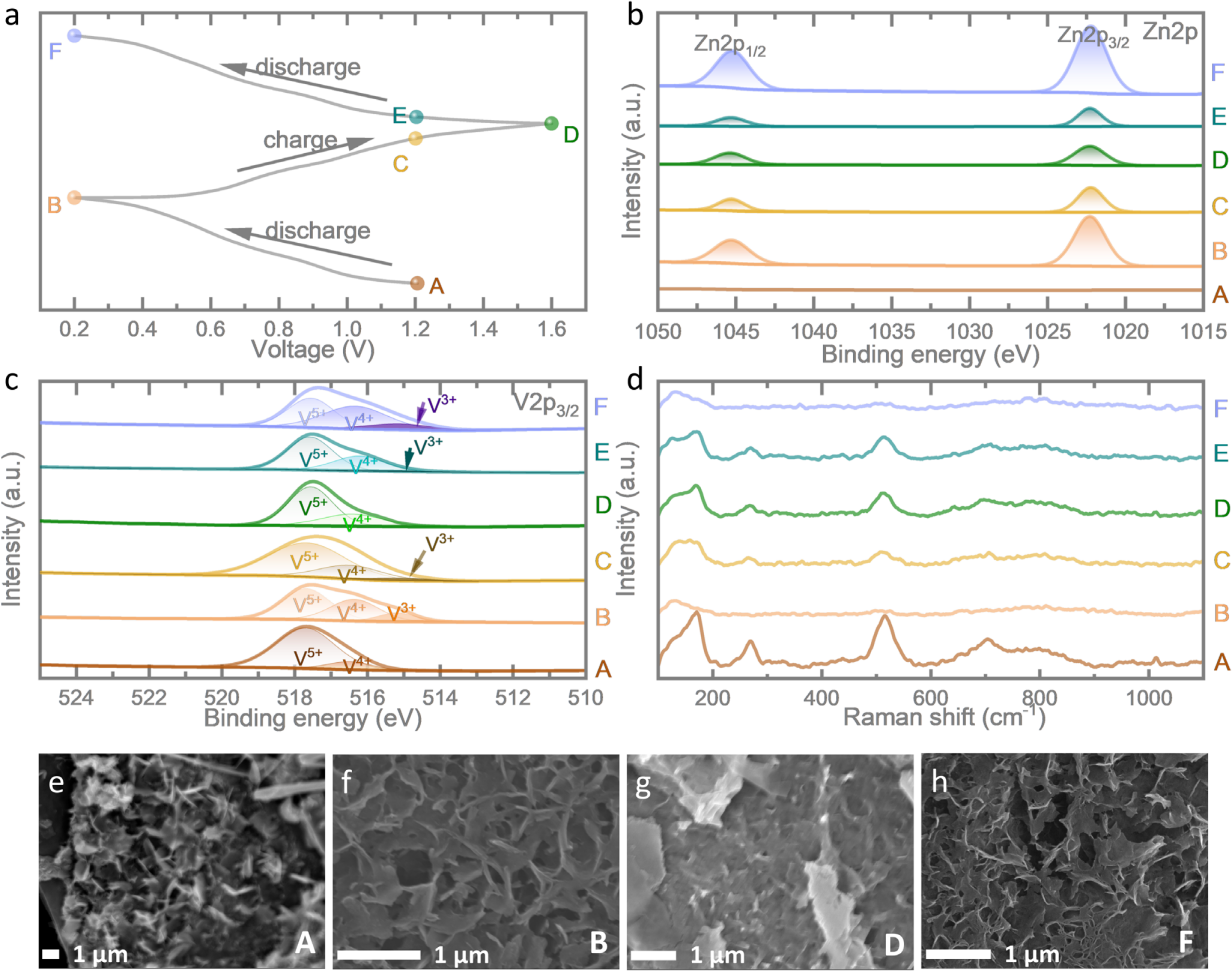
(a) Charge–discharge profiles at 50 µA cm^−^^2^ tested in GG gel electrolyte, (b,c) ex situ high‐resolution XPS spectra and (d) ex situ Raman spectra of K_x_V_2_O_5_·nH_2_O‐2 cathodes at different states of charge. (e–h) Corresponding SEM images of K_x_V_2_O_5_·nH_2_O‐2 cathodes.

To further elucidate the cooperative H^+^/Zn^2^^+^ co‐storage mechanism in these Zn‐TFBs, six K_x_V_2_O_5_·nH_2_O‐2 cells were discharged and charged to different states of charge (points A–F), after which the cathodes were disassembled, cleaned, and subjected to ex situ characterizations (Figure 4a). High‐resolution Zn 2p and V 2p_3_/_2_ XPS spectra are shown in Figure 4b,c. At the pristine state (point A), no Zn 2p signals are detected. Upon discharging to 0.2 V (point B), clear Zn 2p_1_/_2_ and Zn 2p_3_/_2_ peaks appear at 1045.3 and 1022.3 eV, confirming the successful intercalation of Zn^2^^+^ into the K_x_V_2_O_5_·nH_2_O lattice [58, 59]. These peaks intensities decrease during charging to 1.6 V (point D), consistent with Zn^2^^+^ deintercalation, and reappear with strong intensity at point F in the second discharge, verifying the reversibility of the process. Zn^2^^+^ intercalation/deintercalation is also reflected in the evolution of the V oxidation states. At point A, only V^5^^+^ and V^4^^+^ components are present. After full discharge to 0.2 V, the V^5^^+^ signal decreases while V^4^^+^ increases, indicating partial reduction of V^5^^+^ to V^4^^+^. A new V^3^^+^ peak emerges at 515.1 eV, showing further reduction of V^4^^+^ to V^3^^+^ [60]. Upon charging to 1.6 V, the V^3^^+^ peak disappears, the V^4^^+^ signal weakens, and the V^5^^+^ peak intensifies, signifying stepwise oxidation from V^3^^+^ → V^4^^+^ → V^5^^+^. During the second discharge, the peak evolution from points E to F follows the same trend as from A to B, with point F displaying an XPS state identical to point B. These observations confirm that K_x_V_2_O_5_·nH_2_O‐2 Zn‐TFBs operate via a reversible Zn^2^^+^ insertion/extraction mechanism along with cooperative H^+^‐storage [58, 59]. Ex‐situ Raman spectra (Figure 4d) provide further insight. During discharge, Zn^2^^+^ insertion disrupts the layered structure, leading to decreased intensities of Raman bands between 100–600 cm^−^^1^. In contrast, intensities recover during charging as Zn^2^^+^ ions are released [45]. Notably, the V–O–V bending vibration peak at 156 cm^−^^1^ flattens upon discharge, suggesting that interlayer Zn^2^^+^ restricts lattice bending vibrations. This peak sharpens again during charging, confirming the reversible structural response. The subsequent discharge reproduces the same distortions, indicating a restorable Zn^2^^+^ storage mechanism. SEM images of cathodes at states A, B, D, and F (Figure 4e–h) show consistent flake‐like morphologies, demonstrating that the microstructure of K_x_V_2_O_5_·nH_2_O remains stable during repeated cooperative H^+^/Zn^2^^+^ co‐storage intercalation/deintercalation.

**FIGURE 5:**
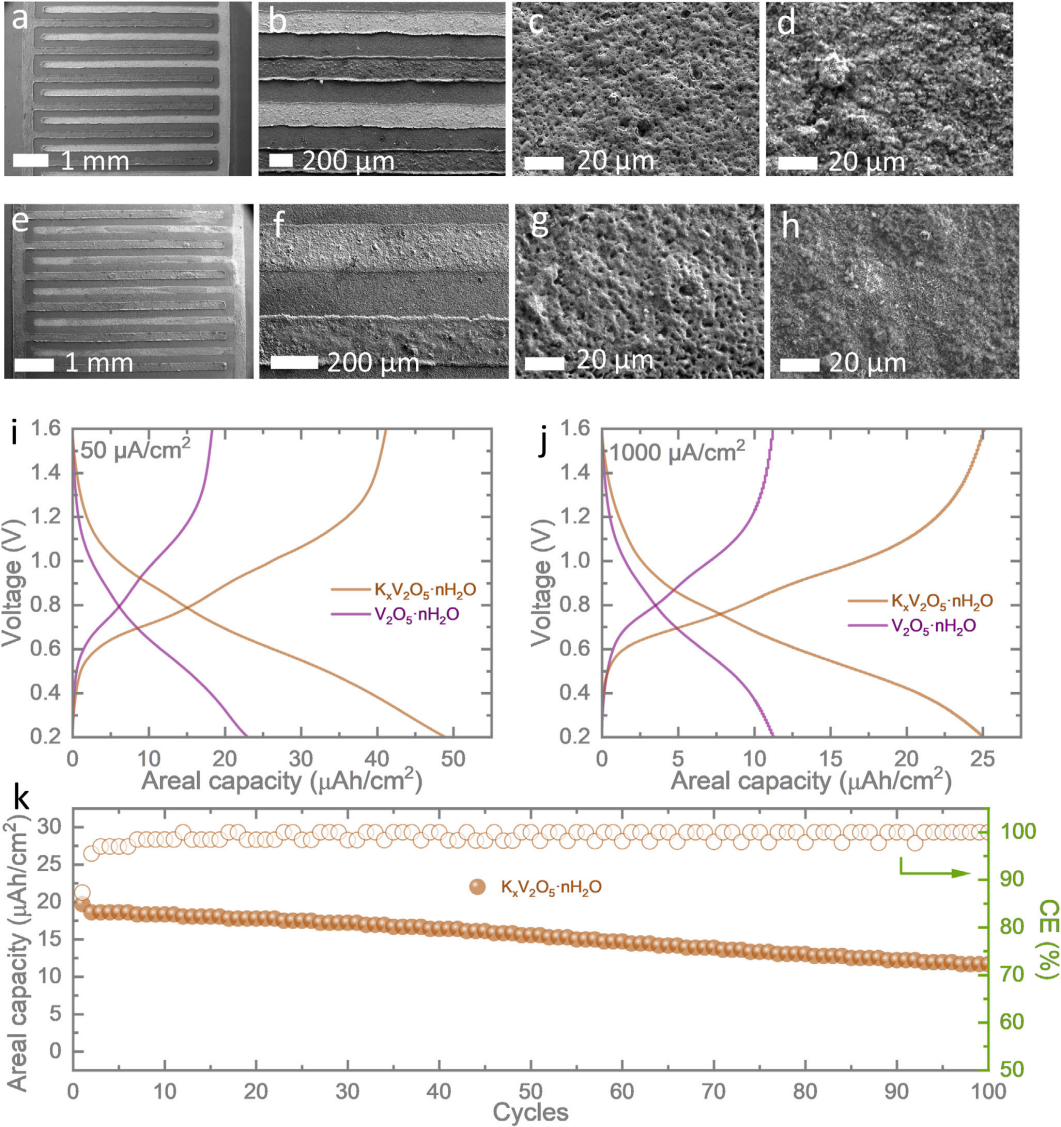
SEM images of (a–d) K_x_V_2_O_5_·nH_2_O and (e–h) V_2_O_5_·nH_2_O Zn‐MBs at different magnifications. Comparative GDC profiles at (i) 50 and (j) 1000 µA cm^−^^2^, and (k) long‐term cycling performance of K_x_V_2_O_5_·nH_2_O Zn‐MB at 500 µA cm^−^^2^.

Next, V_2_O_5_·nH_2_O and K_x_V_2_O_5_·nH_2_O were electrodeposited onto Au IDEs, while Zn was deposited onto the opposite IDEs to fabricate K_x_V_2_O_5_·nH_2_O and V_2_O_5_·nH_2_O Zn‐MBs with GG gel electrolyte. SEM images at different magnifications are shown in Figure 5a–h, confirming the successful electrodeposition of Zn (Figure 5d,h), K_x_V_2_O_5_·nH_2_O (Figure 5c), and V_2_O_5_·nH_2_O (Figure 5g) on the IDEs. Comparative CV curves at 0.1 and 0.2 mV s^−^^1^ (Figure S18a,b) reveal two distinct pairs of redox peaks for both devices. Notably, K_x_V_2_O_5_·nH_2_O Zn‐MBs exhibit significantly higher areal currents than V_2_O_5_·nH_2_O Zn‐MBs at both scan rates, indicating improved capacity. This advantage is further validated by GDC tests across current densities from 50 to 1000 µA cm^−^^2^ (Figure S18c,d). The comparative GDC profiles at 50 and 1000 µA cm^−^^2^ are shown in Figure 5i,j. K_x_V_2_O_5_·nH_2_O Zn‐MBs deliver areal capacities of 49 µAh cm^−^^2^ at 50 µA cm^−^^2^ and 25 µAh cm^−^^2^ at 1000 µA cm^−^^2^, more than double those of V_2_O_5_·nH_2_O Zn‐MBs. Long‐term cycling stability, evaluated at 500 µA cm^−^^2^ (Figure 5k and Figure S19a), also highlights the better performance of the K^+^‐intercalated device. K_x_V_2_O_5_·nH_2_O Zn‐MBs begin with an areal capacity of 20 µAh cm^−^^2^, while V_2_O_5_·nH_2_O Zn‐MBs start at 13 µAh cm^−^^2^; after 100 cycles, the measured areal capacities are 12 µAh cm^−^^2^ and 8 µAh cm^−^^2^ for K_x_V_2_O_5_·nH_2_O and V_2_O_5_·nH_2_O Zn‐MBs. SEM images of cycled devices (Figure S19b–i) further confirm that the electrodeposited materials remain well‐adhered to the Au IDEs, without major detachment or structural degradation.

**FIGURE 6:**
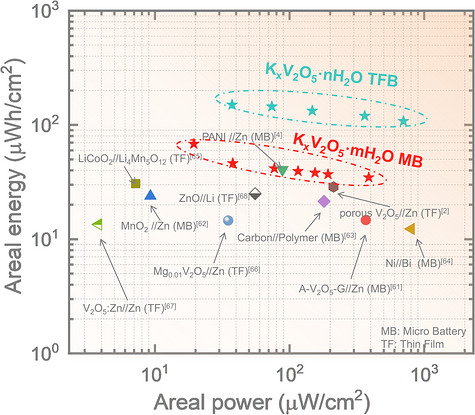
Ragone plots of K_x_V_2_O_5_·nH_2_O Zn‐TFBs and Zn‐MBs, comparing their areal energies and powers with previously reported high‐performance TFBs and MBs: V_2_O_5_//Zn [2], LiCoO_2_//Li_4_Mn_5_O_12_ [65], Mg_0.01_V_2_O_5_//Zn [66], V_2_O_5_:Zn//Zn [67], ZnO//Li, [68] A‐V_2_O_5_‐G//Zn [61], MnO_2_//Zn [62], PANI//Zn [4], Carbon//Polymer [63] and Ni//Bi [64].

The demonstrates the performance in terms of areal energies and areal powers of our K_x_V_2_O_5_·nH_2_O Zn‐MB and K_x_V_2_O_5_·nH_2_O‐2 Zn‐TFB we plot a Ragone plot and compare the performance with reported literature (Figure 6). The areal energies for K_x_V_2_O_5_·nH_2_O Zn‐MB are around 68, 46, 42, 38, 37, 35 µWh cm^−^^2^ at areal powers of 19, 38, 77, 116, 155, 193, 387 µW cm^−^^2^. These performances can exceed many published MBs, such as A‐V_2_O_5_‐G//Zn [61], MnO_2_//Zn [62], PANI//Zn [4], Carbon//Polymer [63], and Ni//Bi [64]. For K_x_V_2_O_5_·nH_2_O‐2 Zn‐TFB, the areal energies are 150, 145, 133, 120, 108 µWh cm^−^^2^ at areal powers of 37, 74, 147, 362, 701 µW cm^−^^2^, which are much better than most TFBs, for instance, porous V_2_O_5_//Zn [2], LiCoO_2_//Li_4_Mn_5_O_12_ [65], Mg_0.01_V_2_O_5_//Zn [66], V_2_O_5_:Zn//Zn [67], ZnO//Li [68]. To evaluate practical applicability, CV and GDC curves of two K_x_V_2_O_5_·nH_2_O‐2 Zn‐TFBs connected in series or parallel are shown in Figure S20a,b. In the series configuration, the potential window is doubled, while the parallel configuration significantly increases the areal current in CV and the charge–discharge time in GDC. These results demonstrate that by configuring the devices in series or parallel, different output voltages, discharge times, and currents can be achieved. For instance, two serially connected K_x_V_2_O_5_·nH_2_O‐2 Zn‐TFBs successfully powered a red LED (Figure S20c) and operated an indoor/outdoor thermometer with a hygrometer clock for 45 min (Figure S20d–f).

## 3 Conclusions

In conclusion, this study uncovers a fundamentally new charge‐storage mechanism in K^+^‐pre‐intercalated V_2_O_5_·nH_2_O cathodes for Zn‐TFBs and Zn‐MBs. Breaking away from the conventional interlayer‐expansion paradigm, we demonstrate that K^+^ incorporation induces a subtle interlayer contraction while simultaneously triggering profound electronic and defect‐chemical transformations, including the generation of abundant oxygen vacancies, the stabilization of mixed‐valence V^4^^+^/V^5^^+^ centers, and a marked enhancement in electronic conductivity. These coupled effects activate highly efficient proton‐Zn^2^^+^ cooperative charge storage, dramatically accelerating ion diffusion, facilitating electron transport, and increasing the density of electrochemically accessible redox sites. Comprehensive electrochemical analyses, supported by ex situ structural and spectroscopic characterization, confirm a highly reversible Zn^2^^+^/H^+^ insertion‐extraction process and a robust cathode microstructure, while impedance spectroscopy reveals substantially reduced charge‐transfer resistance and faster ion‐transport kinetics in K^+^‐engineered electrodes. As a result, the K_x_V_2_O_5_·nH_2_O cathodes consistently deliver more than two‐fold higher areal capacities, superior rate capability, enhanced cycling durability, and outstanding self‐discharge resistance in Zn‐TFBs compared with pristine V_2_O_5_·nH_2_O. By establishing defect‐mediated proton‐Zn^2^^+^ co‐storage as the dominant mechanism governing performance, this work elevates K^+^ pre‐intercalation from a simple structural modifier to a powerful tool for lattice, defect, and valence‐state engineering. The insights presented here provide a new blueprint for designing next‐generation, binder‐free, high‐energy, and high‐power Zn‐TFBs and Zn‐MBs, accelerating their deployment in wearable, flexible, and on‐chip electronic systems.

## Author Contributions

**Jingli Luo**: conceptualization, investigation, methodology, validation, visualization, writing – review and editing, writing – original draft, data curation, formal analysis. **Sanat Nalini Paltasingh**: data curation, formal analysis, writing – original draft, writing – review and editing, validation, methodology. **Narayan Bastola**: data curation, formal analysis, writing – review and editing. **Yijia Zhu**: data curation, formal analysis, writing – review and editing. **Debashish Das**: data curation, formal analysis, writing – review and editing. **Shuhui Li**: data curation, formal analysis, writing – review and editing. **Firoz Alam**: writing – review and editing, data curation. **Subhra R. Pattanayak**: data curation, formal analysis, writing – review and editing. **Sijin Liu**: data curation, writing – review and editing. **Tharangattu N. Narayanan**: writing – review and editing. **Georgios Nikiforidis**: writing – review and editing. **Gopinathan Sankar**: writing – review and editing. **Saroj Kumar Nayak**: writing – review and editing, software. **Buddha Deka Boruah**: conceptualization, software, supervision, resources, project administration, writing – review and editing, writing – original draft, funding acquisition.

## Conflicts of Interest

The authors declare no conflicts of interest.

## Supporting information




**Supporting File**: advs75851‐sup‐0001‐SuppMat.pdf.

## Data Availability

The data that support the findings of this study are available from the corresponding author upon reasonable request.

## References

[1] Y. Yang and W. Gao , “Wearable and Flexible Electronics for Continuous Molecular Monitoring,” Chemical Society Reviews 48 (2019): 1465–1491, 10.1039/C7CS00730B. 29611861

[2] J. Luo , M. Cao , N. Naresh , et al., “Chemically Processed Porous V_2_O_5_ Thin‐Film Cathodes for High‐Performance Thin‐Film Zn‐Ion Batteries,” Advanced Functional Materials 34 (2024): 2417607.

[3] P. Li , M. Liao , J. Li , et al., “Rechargeable Micro‐Batteries for Wearable and Implantable Applications,” Small Structures 3 (2022): 2200058, 10.1002/sstr.202200058.

[4] S. Bi , F. Wan , S. Huang , X. Wang , and Z. Niu , “A Flexible Quasi‐Solid‐State Bifunctional Device with Zinc‐Ion Microbattery and Photodetector,” ChemElectroChem 6 (2019): 3933–3939, 10.1002/celc.201900966.

[5] M. Wakihara , “Recent Developments in Lithium Ion Batteries,” Materials Science and Engineering: R: Reports 33 (2001): 109–134, 10.1016/S0927-796X(01)00030-4.

[6] S.‐H. Cho , J.‐S. Park , J. H. Kim , et al., “Oxygen‐Related Defect Engineering of Amorphous Vanadium Pentoxide Cathode for Achieving High‐Performance Thin‐Film Aqueous Zinc‐Ion Batteries,” ACS Applied Energy Materials 6 (2023): 2719–2727, 10.1021/acsaem.2c03055.

[7] C. A. F. Nason and Y. Xu , “Pre‐Intercalation: A Valuable Approach for the Improvement of Post‐Lithium Battery Materials,” eScience 4 (2024): 100183, 10.1016/j.esci.2023.100183.

[8] C. Qiu , H. Huang , M. Yang , et al., “Advancements in Layered Cathode Materials for Next‐Generation Aqueous Zinc‐Ion Batteries: A Comprehensive Review,” Energy Storage Materials 72 (2024): 103736, 10.1016/j.ensm.2024.103736.

[9] S. W. D. Gourley , R. Brown , B. D. Adams , and D. Higgins , “Zinc‐ion Batteries for Stationary Energy Storage,” Joule 7 (2023): 1415–1436, 10.1016/j.joule.2023.06.007.

[10] H. Wang , X. Bi , Y. Bai , et al., “Open‐Structured V_2_O_5_ *n*H_2_O Nanoflakes as Highly Reversible Cathode Material for Monovalent and Multivalent Intercalation Batteries,” Advanced Energy Materials 7 (2017): 1602720, 10.1002/aenm.201602720.

[11] H. Cui , L. Ma , Z. Huang , Z. Chen , and C. Zhi , “Organic Materials‐Based Cathode for Zinc Ion Battery,” SmartMat 3 (2022): 565–581.

[12] M. Li , M. Maisuradze , R. Sciacca , I. Hasa , and M. Giorgetti , “A Structural Perspective on Prussian Blue Analogues for Aqueous Zinc‐Ion Batteries,” Batteries & Supercaps 6 (2023): 202300340.

[13] W. Zhang , C. Zuo , C. Tang , et al., “The Current Developments and Perspectives of V_2_O_5_ as Cathode for Rechargeable Aqueous Zinc‐Ion Batteries,” Energy Technology 9 (2020): 2000789, 10.1002/ente.202000789.

[14] V. Mathew , B. Sambandam , S. Kim , et al., “Manganese and Vanadium Oxide Cathodes for Aqueous Rechargeable Zinc‐Ion Batteries: A Focused View on Performance, Mechanism, and Developments,” ACS Energy Letters 5 (2020): 2376–2400, 10.1021/acsenergylett.0c00740.

[15] Y. Qiu , Z. Yan , Z. Sun , et al., “Vanadium Oxide‐Based Cathode Materials for Aqueous Zinc‐Ion Batteries: Energy Storage Mechanism and Design Strategy,” Inorganics 11 (2023): 118, 10.3390/inorganics11030118.

[16] C. Chen , Y. Liu , and L. Li , “Development of Vanadium Oxide in Lithium Ion Batteries,” Journal of Inorganic Materials 19 (2004): 1225–1230.

[17] S. Zhu , S. Chen , H. Zhang , et al., “Vanadium Pentoxide Nanosheets with Rich Oxygen Vacancies as a High‐Performance Electrode for Supercapacitors,” Ionics 28 (2022): 2931–2942, 10.1007/s11581-022-04541-3.

[18] T. Zhou , L. Zhu , L. Xie , et al., “Cathode Materials for Aqueous Zinc‐Ion Batteries: A Mini Review,” Journal of Colloid and Interface Science 605 (2022): 828–850, 10.1016/j.jcis.2021.07.138. 34371427

[19] C. Guan , F. Hu , X. Yu , H.‐L. Chen , G.‐H. Song , and K. Zhu , “High Performance of HNaV_6_O_16_·4H_2_O Nanobelts for Aqueous Zinc‐Ion Batteries with In‐Situ Phase Transformation by Zn(CF_3_SO_3_)_2_ Electrolyte,” Rare Metals 41 (2021): 448–456, 10.1007/s12598-021-01778-1.

[20] H. Wang , X. Bi , Y. Bai , et al., “Open‐Structured V_2_O_5_·*n*H_2_O Nanoflakes as Highly Reversible Cathode Material for Monovalent and Multivalent Intercalation Batteries,” Advanced Energy Materials 7 (2017): 1602720, 10.1002/aenm.201602720.

[21] B. Tian , W. Tang , C. Su , and Y. Li , “Reticular V_2_O_5_ 0.6H_2_O Xerogel as Cathode for Rechargeable Potassium Ion Batteries,” ACS Applied Materials & Interfaces 10 (2017): 642–650, 10.1021/acsami.7b15407. 29256595

[22] Z. Wang , P. Liang , R. Zhang , et al., “Oxygen‐Defective V_2_O_5_ Nanosheets Boosting 3D Diffusion and Reversible Storage of Zinc Ion for Aqueous Zinc‐Ion Batteries,” Applied Surface Science 562 (2021): 150196, 10.1016/j.apsusc.2021.150196.

[23] M. Tian , C. Liu , J. Zheng , et al., “Structural Engineering of Hydrated Vanadium Oxide Cathode by K^+^ Incorporation for High‐Capacity and Long‐Cycling Aqueous Zinc Ion Batteries,” Energy Storage Materials 29 (2020): 9–16, 10.1016/j.ensm.2020.03.024.

[24] A. A. Anisimova , Y. M. Plotnikov , and D. M. Korotin , “Chains of Magnetic Ions in NH^4+^ ‐Intercalated Vanadium Pentoxide,” Physical Review B 111 (2025): 014412, 10.1103/physrevb.111.014412.

[25] K. McColl , I. Johnson , and F. Corà , “Thermodynamics and Defect Chemistry of Substitutional and Interstitial Cation Doping in Layered α‐V_2_O_5_ ,” Physical Chemistry Chemical Physics 20 (2018): 15002–15006, 10.1039/C8CP02187B. 29799043

[26] Z. Li , Y. Ren , L. Mo , et al., “Impacts of Oxygen Vacancies on Zinc Ion Intercalation in VO_2_ ,” ACS Nano 14 (2020): 5581–5589, 10.1021/acsnano.9b09963. 32392033

[27] F. Liu , Z. Chen , G. Fang , et al., “V_2_O_5_ Nanospheres with Mixed Vanadium Valences as High Electrochemically Active Aqueous Zinc‐Ion Battery Cathode,” Nano‐Micro Letters 11 (2019): 25, 10.1007/s40820-019-0256-2. 34137986 PMC7770672

[28] S. Li , X. Wei , H. Chen , et al., “A Mixed‐valent Vanadium Oxide Cathode with Ultrahigh Rate Capability for Aqueous Zinc‐ion Batteries,” Journal of Materials Chemistry A 9 (2021): 22392–22398.

[29] L. Liu , T. Yuan , Z. Li , K. Chen , and W. Huang , Electrochimica Acta 439 (2023): 141717.

[30] Y.‐Q. Li , H. Shi , S.‐B. Wang , et al., “Dual‐phase Nanostructuring of Layered Metal Oxides for High‐Performance Aqueous Rechargeable Potassium Ion Microbatteries,” Nature Communications 10 (2019): 4292, 10.1038/s41467-019-12274-7. PMC6754412 31541111

[31] V. Petkov , P. N. Trikalitis , E. S. Bozin , S. J. L. Billinge , T. Vogt , and M. G. Kanatzidis , “Structure of V_2_O_5_·*n*H_2_O Xerogel Solved by the Atomic Pair Distribution Function Technique,” Journal of the American Chemical Society 124 (2002): 10157–10162, 10.1021/ja026143y. 12188680

[32] T. Yao , Y. Oka , and N. Yamamoto , “Layered Structures of Hydrated Vanadium Oxides. Part 1.—Alkali‐Metal Intercalates A_0.3_V_2_O_5_·*n*H_2_O (A= Na, K, Rb, Cs and NH_4_),” Journal of Materials Chemistry 2 (1992): 331–336.

[33] J. Liu and D. Xue , “Cation‐Induced Coiling of Vanadium Pentoxide Nanobelts,” Nanoscale Research Letters 5 (2010): 1619–1626, 10.1007/s11671-010-9685-z. 21076706 PMC2956034

[34] P. Shvets , K. Maksimova , and A. Goikhman , “In Situ XRD and Raman Study of the Phase Transition in V_2_O_5_ Xerogels,” Journal of Non‐Crystalline Solids 625 (2024): 122751, 10.1016/j.jnoncrysol.2023.122751.

[35] Y. Wang and G. Cao , “Li^+^‐Intercalation Electrochemical/Electrochromic Properties of Vanadium Pentoxide Films by Sol Electrophoretic Deposition,” Electrochimica Acta 51 (2006): 4865–4872, 10.1016/j.electacta.2006.01.026.

[36] G. Du , K. H. Seng , Z. Guo , et al., “Graphene–V_2_O_5_·*n*H_2_O Xerogel Composite Cathodes for Lithium Ion Batteries,” RSC Advances 1 (2011): 690–697.

[37] R. Li , F. Xing , T. Li , et al., “Intercalated Polyaniline in V_2_O_5_ as a Unique Vanadium Oxide Bronze Cathode for Highly Stable Aqueous Zinc Ion Battery,” Energy Storage Materials 38 (2021): 590–598, 10.1016/j.ensm.2021.04.004.

[38] V. Balasubramani , J. Chandrasekaran , R. Marnadu , P. Vivek , S. Maruthamuthu , and S. Rajesh , “Impact of Annealing Temperature on Spin Coated V_2_O_5_ Thin Films as Interfacial Layer in Cu/V_2_O_5_/n‐Si Structured Schottky Barrier Diodes,” Journal of Inorganic and Organometallic Polymers and Materials 29 (2019): 1533–1547, 10.1007/s10904-019-01117-z.

[39] M. Rafique , M. Hamza , M. B. Tahir , S. Muhammad , and A. G. Al‐Sehemi , “Facile Hydrothermal Synthesis of Highly Efficient and Visible Light‐driven Ni‐doped V_2_O_5_ Photocatalyst for Degradation of Rhodamine B Dye,” Journal of Materials Science: Materials in Electronics 31 (2020): 12913–12925.

[40] Z. Cui , X. Dong , Y. Sun , Y. Zhou , Y. Zhang , and F. Dong , “Simultaneous Introduction of Oxygen Vacancies and Bi Metal Onto the {001} Facet of Bi_3_O_4_Cl Woven Nanobelts for Synergistically Enhanced Photocatalysis,” Nanoscale 10 (2018): 16928–16934, 10.1039/C8NR05322G. 30178788

[41] E. A. Konstantinova , A. I. Kokorin , A. A. Minnekhanov , T. V. Sviridova , and D. V. Sviridov , “EPR Study of Photoexcited Charge Carrier Behavior in TiO_2_/MoO‐ and TiO_2_/MoO_3_:V_2_O_5_ Photocatalysts,” Catalysis Letters 149 (2019): 2256–2267, 10.1007/s10562-019-02830-7.

[42] Y. Ba , G. Yang , S. Sun , et al., “K_0.39_V_2_O_5_·0.52H_2_O Nanostructures with Oxygen Vacancies as Cathodes for Aqueous Zinc‐Ion Batteries,” ACS Applied Nano Materials 8 (2025): 1205–1213, 10.1021/acsanm.4c06198.

[43] K. Fang , H. Zhang , P. Chen , et al., “Synergistic Structure Engineering and Electrochemical Activation Modulating Vanadium Oxide Cathode Toward Superior Zinc‐Ion Storage,” Chemical Engineering Journal 496 (2024): 153736, 10.1016/j.cej.2024.153736.

[44] Q. Li , X. Ye , H. Yu , et al., “Pre‐Potassiated Hydrated Vanadium Oxide as Cathode for Quasi‐Solid‐State Zinc‐Ion Battery,” Chinese Chemical Letters 33 (2022): 2663–2668, 10.1016/j.cclet.2021.09.091.

[45] C. Wang , W. Xie , H. Cao , et al., “In Situ Raman Observation of Zinc‐Induced Structural Dynamics and Charge Transfer of a Layered V_2_O_5_ ,” Journal of The Electrochemical Society 170 (2023): 083511, 10.1149/1945-7111/acf243.

[46] C. L. Londoño‐Calderón , C. Vargas‐Hernandez , and J. F. Jurado , “Desorption Influence of Water on Structural, Electrical Properties and Molecular Order of Vanadium Pentoxide Xerogel Films,” Revista Mexicana de Fisica 56 (2010): 411–415.

[47] N. Zhang , Y. Dong , M. Jia , et al., “Rechargeable Aqueous Zn–V_2_O_5_ Battery with High Energy Density and Long Cycle Life,” ACS Energy Letters 3 (2018): 1366–1372, 10.1021/acsenergylett.8b00565.

[48] T. Pian , N. Wang , Z. Huang , et al., “Grotthuss Topochemistry Realizes Ah‐Level Fast‐Chargeable Zinc‐Vanadium Pouch Battery,” Advanced Functional Materials 36 (2025): 16731, 10.1002/adfm.202516731.

[49] L. Wang , J. Yan , Y. Hong , Z. Yu , J. Chen , and J. Zheng , “Ultrahigh‐Rate and Ultralong‐Life Aqueous Batteries Enabled by Special Pair‐dancing Proton Transfer,” Science Advances 9 (2023): adf4589, 10.1126/sciadv.adf4589. PMC10162668 37146149

[50] M. Li , S. Li , D. Yan , Y. Ma , X. Niu , and L. Wang , “Electrolyte Design Weakens Lithium‐Ion Solvation for a Fast‐charging and Long‐Cycling Si Anode,” Chemical Science 16 (2025): 2609–2618, 10.1039/D4SC08125K. 39811004 PMC11728059

[51] H. Zhou , G. Zhu , S. Dong , et al., “Ethanol‐Induced Ni^2+^ ‐Intercalated Cobalt Organic Frameworks on Vanadium Pentoxide for Synergistically Enhancing the Performance of 3D‐Printed Micro‐Supercapacitors,” Advanced Materials 35 (2023): 2211523, 10.1002/adma.202211523. 36807415

[52] C. Gao , J. Huang , Y. Xiao , et al., “A Seamlessly Integrated Device of Micro‐supercapacitor and Wireless Charging with Ultrahigh Energy Density and Capacitance,” Nature Communications 12 (2021): 2647, 10.1038/s41467-021-22912-8. PMC8113435 33976170

[53] L. Ye , L. Lou , H. Shi , et al., “Alleviating Self‐Discharge of Printed Interdigital Supercapacitor Based on Conjugatedly Configured Pairs of Pre‐sodiated Manganese Oxide,” Chemical Engineering Journal 504 (2025): 158996, 10.1016/j.cej.2024.158996.

[54] Y. Wang , Y.‐Z. Zhang , Y.‐Q. Gao , G. Sheng , and J. E. ten Elshof , “Defect Engineering of MnO_2_ Nanosheets by Substitutional Doping for Printable Solid‐State Micro‐Supercapacitors,” Nano Energy 68 (2020): 104306, 10.1016/j.nanoen.2019.104306.

[55] J. Chung , H. Park , and C. Jung , “Electropolymerizable Isocyanate‐Based Electrolytic Additive to Mitigate Diffusion‐Controlled Self‐Discharge for Highly Stable and Capacitive Activated Carbon Supercapacitors,” Electrochimica Acta 369 (2021): 137698, 10.1016/j.electacta.2020.137698.

[56] M. F. El‐Kady and R. B. Kaner , “Scalable Fabrication of High‐Power Graphene Micro‐Supercapacitors for Flexible and On‐Chip Energy Storage,” Nature Communications 4 (2013): 1475, 10.1038/ncomms2446. 23403576

[57] W. Gao , N. Singh , L. Song , et al., “Direct Laser Writing of Micro‐Supercapacitors on Hydrated Graphite Oxide Films,” Nature Nanotechnology 6 (2011): 496–500, 10.1038/nnano.2011.110. 21804554

[58] J. Wu , Z. Yang , H. Chen , L. Deng , Y. Rong , and Z. Fu , “In‐situ Prepared of Quadrilateral Flake Zn_0.25_(NH_4_)V_2_O_5_H_2_O as a Cathode for Aqueous Rechargeable Zn‐Ion Batteries,” Applied Surface Science 592 (2022): 153137, 10.1016/j.apsusc.2022.153137.

[59] R. Dong , T. Zhang , J. Liu , et al., “Mechanistic Insight into Polypyrrole Coating on V_2_O_5_ Cathode for Aqueous Zinc‐Ion Battery,” ChemElectroChem 9 (2022): 202101441, 10.1002/celc.202101441.

[60] Y. Ran , P. Hong , J. Ren , et al., “V_2_O_5_/NaV_6_O_15_ Nanocomposites Synthesized by Molten Salt Method as a High‐Performances Cathode Material for Aqueous Zinc‐Ion Batteries,” Nanotechnology 33 (2021): 115402, 10.1088/1361-6528/ac3fe1. 34874293

[61] X. Wang , Y. Li , S. Wang , et al., “2D Amorphous V_2_O_5_/Graphene Heterostructures for High‐Safety Aqueous Zn‐Ion Batteries with Unprecedented Capacity and Ultrahigh Rate Capability,” Advanced Energy Materials 10 (2020): 2000081, 10.1002/aenm.202000081.

[62] X. Wang , S. Zheng , F. Zhou , et al., “Scalable Fabrication of Printed Zn//MnO2 Planar Micro‐Batteries with High Volumetric Energy Density and Exceptional Safety,” National Science Review 7 (2019): 64–72, 10.1093/nsr/nwz070. 34692018 PMC8288951

[63] H.‐S. Min , B. Y. Park , L. Taherabadi , et al., “Fabrication and Properties of a Carbon/Polypyrrole Three‐Dimensional Microbattery,” Journal of Power Sources 178 (2008): 795–800, 10.1016/j.jpowsour.2007.10.003.

[64] L. He , T. Hong , X. Hong , et al., “Ultrastable High‐Energy on‐Chip Nickel–Bismuth Microbattery Powered by Crystalline Bi Anode and Ni–Co Hydroxide Cathode,” Energy Technology 7 (2019): 1900144, 10.1002/ente.201900144.

[65] M. Kotobuki , Y. Suzuki , H. Munakata , et al., “Effect of Sol Composition on Solid Electrode/Solid Electrolyte Interface for All‐Solid‐State Lithium Ion Battery,” Electrochimica Acta 56 (2011): 1023–1029, 10.1016/j.electacta.2010.11.008.

[66] X. Luo , H. Xu , Y. He , et al., “Layered Magnesium Vanadate Film Electrodes as High‐Performance Cathode Materials for Thin‐Film Non‐Aqueous Zinc‐ion Batteries,” Journal of Electroanalytical Chemistry 967 (2024): 118443, 10.1016/j.jelechem.2024.118443.

[67] Y. Zhang , H. Xu , Y. He , et al., “Zn‐Doped V_2_O_5_ Film Electrodes as Cathode Materials for High‐Performance Thin‐Film Zinc‐Ion Batteries,” Solid State Ionics 416 (2024): 116658, 10.1016/j.ssi.2024.116658.

[68] N. Garino , A. Lamberti , R. Gazia , A. Chiodoni , and C. Gerbaldi , “Cycling Behaviour of Sponge‐Like Nanostructured ZnO as Thin‐film Li‐Ion Battery Anodes,” Journal of Alloys and Compounds 615 (2014): S454–S458, 10.1016/j.jallcom.2013.11.157.

