# Supplementary material for: K+‐Triggered Defect Engineering and Proton‐Coupled Storage in V2O5·nH2O for Advanced Zn‐Ion Thin‐Film and Microbatteries
Source: Adv Sci (Weinh). 2026 May 29:e75851. Online ahead of print. doi: 10.1002/advs.75851 (PMC13335772; doi:10.1002/advs.75851)
Supplement: Supplementary file 1 — Supporting File: advs75851‐sup‐0001‐SuppMat.pdf. [file ADVS-9999-e75851-s001.pdf]

Supporting Information

for

K^+-Triggered Defect Engineering and Proton-Coupled Storage in $V_2O_5 \cdot nH_2O$ for Advanced Zn-Ion Thin-Film and Micro-Batteries

Jingli Luo,[1] Sanat Nalini Paltasingh,[2] Narayan Bastola,[3] Yijia Zhu,[1] Debashish Das,[2] Shuhui Li,[4] Firoz Alam,[5] Subhra R. Pattanayak,[6] Sijin Liu,[1] Tharangattu N. Narayanan,[6] Georgios Nikiforidis,[1] Gopinathan Sankar,[4] Ivan P Parkin,[4] Saroj Kumar Nayak,[2,*] Buddha Deka Boruah[1,*]

[1]Institute for Materials Discovery, University College London, London WC1E 7JE, United Kingdom

[2]School of Basic Sciences, Indian Institute of Technology Bhubaneswar, Khordha, Odisha, 752050, India

[3]Department of Mechanical Engineering, University of Bath, Bath, BA2 2ET, UK

[4]Department of Chemistry, University College London, London, WC1H 0AJ United Kingdom

[5]Department of Electronic and Electrical Engineering, University College London, London, WC1E 6BT, UK

[6]Tata Institute of Fundamental Research Hyderabad, Serilingampally Mandal, Hyderabad 500046, India.

Experimental Section

Materials: vanadium(IV) oxide sulfate hydrate (Aldrich, 97%), potassium sulfate (Sigma-Aldrich, ≧99.0%), sulfuric acid (Sigma-Aldrich, 95.0-98.0%), sodium sulfate anhydrous (Thermo Scientific, 99%), zinc sulfate heptahydrate (Thermo Scientific, 98%), boric acid (Sigma-Aldrich, ≧99.5%), zinc trifluoromethanesulfonate (Fluorochem), guar gum (Sigma-Aldrich), vanadium(V) oxide (Sigma-Aldrich, ≧98%).

Preparation of $K_xV_2O_5·nH_2O$, $V_2O_5·nH_2O$ cathodes for coin cells and Zn-TFBs: The electrodeposition solution for $V_2O_5·nH_2O$ cathode was made by dissolving 4.1 g VOSO4 powder in 250 mL deionized water, adjusting the PH to 1.8 with diluted sulfuric acid. Just add extra 1.3 g K_2SO_4 powder before changing PH level for preparing $K_xV_2O_5·nH_2O$ precursor solution. All electrodeposition process were carried out with three-electrode set-up (Vionic, Ω Metrohm), graphene paper, one side covered to inhibit double-side electrodeposition, was held by a Pt clip to constitute a working electrode, a Pt plate and an aqueous Ag/AgCl electrode were used as counter electrode and reference electrode. After being electrodeposited at 4 mA/cm^2 for 2h or 4h and removing the covering, deposited graphene paper was heated in Muffle furnace at 200 °C for 12h. Then cut the product into 1 cm × 1 cm cathodes. The mass loading of both $V_2O_5·nH_2O$ and $K_xV_2O_5·nH_2O$ are around 0.64 mg cm^{-2} for 2h-electrodeposition and 1.06 mg cm^{-2} for 4h-electrodeposition.

Preparation of $K_xV_2O_5·nH_2O$, $V_2O_5·nH_2O$ cathodes and Zn anodes for Zn-MBs: The electrolyte recipes of cathode electrodeposition for Zn-MBs were the same as the ones for Zn-TFBs. For Zn electrodeposition, the precursor solution was prepared by dissolving 12.5 g Na_2SO_4, 22.3 g $ZnSO_4·7H_2O$, 2 g boric acid in 91 ml deionized water. Three-electrode setup was still used, but the graphene paper was replaced by a commercial Au integrated patterned chip, and a Pt wire were used as counter electrode. Cathode material was firstly electrodeposited on the chip at 200 mA/cm^2 for 20 min. Then Zn anode was electrodeposited on the other side of the chip at -40 mA/cm^2 for 3.5 min.

Preparation of commercial V_2O_5 cathodes for coin cells: Commercial V_2O_5 powder was

dissolved in deionized water to get V_2O_5 mixture. The mixture was blade casted on graphene paper and then heated in 200°C Muffle furnance for 12h. Eventually, also cut the product into 1 cm × 1 cm cathodes.

Electrolytes: Aqueous 3 M $Zn(CF_3SO_3)_2$ electrolyte was prepared by adding 5.4530 g $Zn(CF_3SO_3)_2$ salt in 5 ml deionized water. To get guar gum (GG) gel electrolyte, 0.0526 g GG was slowly added in above aqueous electrolyte while continuous stirring. Then the mixture needed to be stirred for additional 15 min to get the gel-like electrolyte. Aqueous H_2SO_4 electrolyte was prepared by adding H_2SO_4 slowly in water till the solution shows its PH as 3.98, which is the same as the aqueous 3 M $Zn(CF_3SO_3)_2$ electrolyte.

Material characterizations: The morphology of samples were displayed by scanning electron microscopy (SEM, Zeiss EVO LS15). The microstructures and elemental compositions of samples were exhibited by transmission electron microscopy (TEM, JEOL JEM-2100) and combined energy dispersive spectroscopy (EDS). The phase structure of samples were indicated by X-ray diffraction (XRD, AERIS PANalytical Research Edition) machine with Cu Kα radiation. Various vibrational modes of sample molecules were determined with a Raman spectrometer (Renishaw inVia™ confocal Raman Microscope), adopting a laser wavelength of 515 nm. The water contents of samples were figured out through thermogravimetric analysis (TGA, PerkinElmer Spectrum TGA 4000) at temperature range from 30°C to 500°C with heating rate of 10°C min^{-1}. The optical characteristics of materials were investigated with ultraviolet-visible Spectrometer (UV-vis, Shimadzu UV-1800) over wavelength range of 300-800 nm. The unpaired electrons of samples were studied using electron paramagnetic resonance (EPR, Bruker EMX-micro) with microwave power maintained at 10 mW, having a central field of 340 mT. The field modulation frequency was set at 100 kHz, with modulation amplitude of 0.4 mT. The elemental compositions, valence states and molecular structure of samples were analyzed via X-ray photoelectron spectroscopy (XPS, ThermoFisher Scientific) with monochromatic Al Kα X-rays (1486.6 eV), using a pass energy of 50 eV for narrow spectra and 200 eV for wide spectra. The viscosity of aqueous electrolyte and gel electrolyte were detected by viscometer (Brookfield CAP 2000+) at 50°C.

Computational details: First-principles calculations were performed using Density Functional Theory (DFT)[1-3], utilizing the Vienna Ab Initio Simulation Package (VASP)[4, 5] code for our study. The spin-polarized generalized gradient approximation[6] of Perdew-Burke-Ernzerhof[7] (GGA-PBE+U) is used to describe the exchange correlation potential. Hubbard parameters are used to treat the 3d orbital electrons of V with a value of U_d=3 eV. A plane-wave cutoff energy of 520 eV was established for all calculations. All geometrical structures were optimized by fully relaxing atomic positions until the Hellman-Feynman forces on each atom were reduced below a threshold of 0.01 eV, complying with a total energy convergence criterion of 1×10^{-5} eV. Given that layered systems are heavily influenced by van der Waals interactions, the DFT-D3 method[8] was applied to correct for potential energy and interatomic forces. For structure optimization, the Brillouin zone was sampled using gamma-centered k-point grids of 2×2×1.

Input file for the crystal structure of double layered slab of orthorhombic V_2O_5

```
Double layered V2O5
1.0
    3.5517498100000000     0.0000000000000000     0.0000000000000002
   -0.0000000000000003     4.2633577899999997     0.0000000000000003
    0.0000000000000000     0.0000000000000000    11.6044171100000000
V O
4 10
direct
    0.0000000000000000     0.8885870400000000     0.1482996500000000 V5+
    0.5000000000000000     0.1114129600000000     0.3517003499999991 V5+
    0.5000000000000000     0.1114129600000000     0.6482996500000000 V5+
    0.0000000000000000     0.8885870400000000     0.8517003500000000 V5+
    0.0000000000000000     0.9936429700000000     0.0000000000000000 O2-
    0.0000000000000000     0.5139977200000000     0.1466839999999990 O2-
    0.5000000000000000     0.9941575600000000     0.1818999900000000 O2-
    0.0000000000000000     0.0058424400000000     0.3181000099999990 O2-
    0.5000000000000000     0.4860022800000000     0.3533159999999990 O2-
    0.5000000000000000     0.0063570299999990     0.5000000000000000 O2-
    0.5000000000000000     0.4860022800000000     0.6466839999999990 O2-
```

0.0000000000000000	0.0058424400000000	0.6818999900000000	O2-
0.5000000000000000	0.9941575600000000	0.8181000100000000	O2-
0.0000000000000000	0.5139977200000000	0.8533160000000011	O2-

Input file for the crystal structure of a bilayered sheet of orthorhombic V_2O_5

Bilayered Sheet of V_2O_5

1.000

11.7220001221000008	0.0000000000000000	0.0000000000000000
0.0000000000000000	3.5699999332000001	0.0000000000000000
-0.2714077562000000	0.0000000000000000	38.5168034681999956

V O

16 40

Cartesian

3.2143591020000000	0.0000000000000000	27.9161582579999994	V1
8.2362332100000000	0.0000000000000000	24.9827656910000009	V2
9.0753595120000004	1.7849999670000001	27.9161582579999994	V3
2.3752329740000002	1.7849999670000001	24.9827656910000009	V4
6.6262485570000003	0.0000000000000000	27.8827594179999991	V5
4.8243437589999996	0.0000000000000000	25.0161642250000007	V6
0.7652482340000000	1.7849999670000001	27.8827594179999991	V7
10.6853441700000005	1.7849999670000001	25.0161642250000007	V8
3.2143591020000000	0.0000000000000000	16.4161582579999994	V9
8.2362332100000000	0.0000000000000000	13.4827656910000009	V10
9.0753595120000004	1.7849999670000001	16.4161582579999994	V11
2.3752329740000002	1.7849999670000001	13.4827656910000009	V12
6.6262485570000003	0.0000000000000000	16.3827594179999991	V13
4.8243437589999996	0.0000000000000000	13.5161642250000007	V14
0.7652482340000000	1.7849999670000001	16.3827594179999991	V15
10.6853441700000005	1.7849999670000001	13.5161642250000007	V16
1.1968582940000001	0.0000000000000000	27.5741091370000007	O1
10.2537340340000007	0.0000000000000000	25.3248135900000015	O2
7.0578587040000000	1.7849999670000001	27.5741091370000007	O3
4.3927339730000003	1.7849999670000001	25.3248135900000015	O4
3.4230615879999999	0.0000000000000000	29.4559548319999998	O5
8.0275307300000005	0.0000000000000000	23.4429686589999982	O6
9.2840612999999994	1.7849999670000001	29.4559548319999998	O7
2.1665303190000000	1.7849999670000001	23.4429686589999982	O8
4.9900597030000000	0.0000000000000000	27.4762163249999993	O9
6.4605322750000003	0.0000000000000000	25.4227064770000020	O10

10.8510597640000004	1.7849999670000001	27.4762163249999993	O11
0.5995322140000000	1.7849999670000001	25.4227064770000020	O12
6.9068128499999997	0.0000000000000000	29.4571061890000010	O13
4.5437794680000003	0.0000000000000000	23.4418173020000005	O14
1.0458125270000000	1.7849999670000001	29.4571061890000010	O15
10.4047798789999995	1.7849999670000001	23.4418173020000005	O16
8.7302694410000008	0.0000000000000000	27.1399256720000004	O17
2.7203223639999998	0.0000000000000000	25.7589969780000025	O18
2.8692699039999998	1.7849999670000001	27.1399256720000004	O19
8.5813219010000008	1.7849999670000001	25.7589969780000025	O20
1.1968582940000001	0.0000000000000000	16.0741091370000007	O21
10.2537340340000007	0.0000000000000000	13.8248135899999998	O22
7.0578587040000000	1.7849999670000001	16.0741091370000007	O23
4.3927339730000003	1.7849999670000001	13.8248135899999998	O24
3.4230615879999999	0.0000000000000000	17.9559548319999998	O25
8.0275307300000005	0.0000000000000000	11.9429686589999999	O26
9.2840612999999994	1.7849999670000001	17.9559548319999998	O27
2.1665303190000000	1.7849999670000001	11.9429686589999999	O28
4.9900597030000000	0.0000000000000000	15.9762163249999993	O29
6.4605322750000003	0.0000000000000000	13.9227064770000002	O30
10.8510597640000004	1.7849999670000001	15.9762163249999993	O31
0.5995322140000000	1.7849999670000001	13.9227064770000002	O32
6.9068128499999997	0.0000000000000000	17.9571061890000010	O33
4.5437794680000003	0.0000000000000000	11.9418173020000005	O34
1.0458125270000000	1.7849999670000001	17.9571061890000010	O35
10.4047798789999995	1.7849999670000001	11.9418173020000005	O36
8.7302694410000008	0.0000000000000000	15.6399256720000004	O37
2.7203223639999998	0.0000000000000000	14.2589969780000008	O38
2.8692699039999998	1.7849999670000001	15.6399256720000004	O39
8.5813219010000008	1.7849999670000001	14.2589969780000008	O40

Coin cells, thin-film batteries, microbatteries assembly: CR2032 coin-type cells were adopted for coin cell assembly. Cathode case, $K_xV_2O_5{\cdot}nH_2O$ or $V_2O_5{\cdot}nH_2O$ cathode, Whatman glass microfiber separator, aqueous 3 M $Zn(CF_3SO_3)_2$ electrolyte, zinc anode, spacer, spring, anode case were sequentially placed and then got compressed by crimping machine. Depends on the different precursor solution and electrodeposition time, the corresponded coin cells are named as $K_xV_2O_5{\cdot}nH_2O$-2, $K_xV_2O_5{\cdot}nH_2O$-4, $V_2O_5{\cdot}nH_2O$-2, $V_2O_5{\cdot}nH_2O$-4, respectively. While

analyzing the interaction between proton and commercial V_2O_5, oxygen-deficient $K_xV_2O_5{\cdot}nH_2O$, $V_2O_5{\cdot}nH_2O$ systems, traditional 3 M $Zn(CF_3SO_3)_2$ electrolyte was substituted by aqueous H_2SO_4 electrolyte in commercial V_2O_5, $K_xV_2O_5{\cdot}nH_2O$, $V_2O_5{\cdot}nH_2O$ coin cells. Symmetric cells, containing Zn//Zn and stainless steel//stainless steel (SS//SS) symmetrical cells, were also assembled in CR2032 coin-type cells, but use zinc sheets or SS sheets for both electrodes. Aqueous electrolyte or gel electrolyte was added according to the purpose. For Zn-TFBs assembly, one side of a 1.2 cm × 5 cm graphene paper was initially sticked onto a piece of Kapton tape, the free side was regarded as cathodic conductive part. What above them was prepared 1 cm × 1 cm cathode. A piece of double-side duct tape was cut into a square ring and applied around the cathode. 100 μL GG gel electrolyte was added inside the ring to completely cover the cathode, then followed with Whatman glass microfiber separator and the other 100 μL gel electrolyte. A zinc sheet was previously cut into a 1.2 cm × 1.2 cm square as anode with a 4 cm length of long tail as conductive wire. The anode square was placed upon gel electrolyte with the tail pointed at the opposite direction of cathodic conductive part. Eventually, the other piece of Kapton tape was closely sticked with the first one to create an enclosed space. The assembled Zn-TFBs are separately denoted as $K_xV_2O_5{\cdot}nH_2O$-2, $K_xV_2O_5{\cdot}nH_2O$-4, $V_2O_5{\cdot}nH_2O$-2, $V_2O_5{\cdot}nH_2O$-4 according to the cathode preparation process. For Zn-MBs, two golden conductive bands of the electrodeposited integrated patterned chip were separately connected with a copper wire using conductive silver paste. The chip was put inside a plastic cuvette. Two copper wires were extended out and attached to the outside of the cuvette to prevent shortcut caused by wire contact. Next, 700 μL gel electrolyte was dropped in the cuvette and a slice of parafilm, Kapton tape were used for sealing the device of $K_xV_2O_5{\cdot}mH_2O$ or $V_2O_5{\cdot}mH_2O$ Zn-MBs. All batteries were assembled under ambient environment.

Electrochemical test: Cyclic voltammetry (CV), galvanostatic discharge/charge (GDC) and electrochemical impedance spectroscopy (EIS) were all tested by IviumSoft operator. Long-term cycling was run with NEWARE (BTS4000) system. The CV, GDC, long-term cycling measurements were carried out under a voltage range of 0.2-1.6 V. The scan rates in CV tests were 0.2, 0.4, 0.5, 0.6, 0.8, and 1 mV s^{-1} for coin cells and Zn-TFBs, 0.1, 0.2, 0.4, 0.5, 0.6 mV s^{-1} for Zn-MBs. The areal current in GDC tests were 50, 100, 200, 500, 1000 μA cm^{-2} for general

coin cells and Zn-TFBs, 50, 100, 200, 300, 400, 500, 1000 μA cm^{-2} for Zn-MBs, 500 μA mg^{-1} cm^{-2} for commercial V_2O_5, $K_xV_2O_5{\cdot}nH_2O$, $V_2O_5{\cdot}nH_2O$ coin cells. The areal current in long-term cycling measurements were 1000 μA cm^{-2} for coin cells, Zn-TFBs and 500 μA cm^{-2} for Zn-MBs. EIS was performed at open circuit voltage (OCV, close to 0 V for symmetrical cells) with a frequency range of 0.01 Hz to 100 kHz. EIS spectra for symmetrical cells were obtained at 25°C, 30°C, 35°C, 40°C, 45°C, 50°C, 55°C, 60°C, and 65°C.

Calculations: The band gap energy (E_g, eV) of $K_xV_2O_5{\cdot}nH_2O$ and $V_2O_5{\cdot}nH_2O$ with direct band gap can be obtained from Tauc plot, which is calculated and drawn from UV-vis spectra using Tauc relation (1) shown below:

$$(\alpha \mathrm{h}v)^2 = \mathrm{C}(\mathrm{h} - E_g) \quad (1)$$

Where α is absorption coefficient (cm^{-1}), h is Planck constant (J • s), v is wave frequency (s^{-1}), C is band tailoring constant. h is known as 6.62607015×10^{-34} J • s. After the calculation, Tauc plot can be drawn with photon energy (hv) as x axis, $(\alpha \mathrm{h}v)^2$ as y axis.

Variations in the g-value ($g_e + \delta_g$) suggest subtle changes in the local electronic environment of $K_xV_2O_5{\cdot}nH_2O$ and $V_2O_5{\cdot}nH_2O$, likely influenced by structural defects such as oxygen, thus the numerical value of g can be regarded as a distinctive identifier. The g-parameter, defined through the resonance condition with a equation (2):

$$\mathrm{h}v_{\mathrm{m}} = (g_e + \delta_g)\beta B \quad (2)$$

Where v_{m} is microwave frequency (Hz), g_e is the g-factor of a free electron, δ_g is g-value shift, β is Bohr magneton constant (J T^{-1}), B is resonance magnetic field (T). The value of g_e is 2.0023. The value of β is 9.274×10^{-24}J T^{-1}. v_{m} is set as 9.348 GHz, B is set as 343.75 mT for $K_xV_2O_5{\cdot}nH_2O$, 345.83 mT for $V_2O_5{\cdot}nH_2O$.

The Arrhenius equation (3) can be used to fit activation energies (E_a, J mol^{-1}) of 3M $Zn(CF_3SO_3)_2$ electrolyte and GG gel electrolyte from EIS results of their Zn//Zn symmetrical cells in a temperature range (25°C to 65°C):

$$\frac{1}{R_{ct}} = Ae^{\frac{-E_a}{RT}} \tag{3}$$

Where R_{ct} is charge-transfer resistance (Ohm) read from the Nyquist plots of symmetrical cell, A is frequency factor (Ohm^{-1}), R is idea gas constant (J mol^{-1} K^{-1}), T is temperature (K). R is known as 8.3145 J mol^{-1} K^{-1}, based on different T and the corresponding R_{ct}, E_a can be fitted.

The formula (4) below can be used to calculate ionic conductivity (λ) of two electrolytes from EIS results of their SS//SS symmetrical cells:

$$\lambda = \frac{L}{R_e \times S} \tag{4}$$

Where L is the thickness of separator (cm), R_e is electrolyte resistance (Ohm), S is the contact area between SS and electrolyte (cm^2). In this project, the separator made of glass fiber has the L of 260 μm, the totally contacted SS sheet has the S of 1 cm^2. λ of two electrolyte at specific temperature can be calculated.

Following two relations (5, 6) can be used to compare the diffusion coefficient for Zn^{2+} ($D_{zn^{2+}}$, cm^2 s^{-1}) in $K_xV_2O_5{\cdot}nH_2O$-2 Zn-TFB and $V_2O_5{\cdot}nH_2O$-2 Zn-TFB, from low frequency region of their Nyquist plots:

$$Re(Z) = (R_e + R_{ct}) + \frac{\sigma}{\omega^{0.5}} \tag{5}$$

$$D_{zn^{2+}} = \frac{R^2T^2}{2{S_a}^2n^4F^4C^2\sigma^2} = \frac{K}{\sigma^2} \tag{6}$$

Where $Re(Z)$ is real impedance (Ohm) of the Nyquist plot, R_e and R_{ct} can be read from the Nyquist plot, σ is Warburg coefficient (Ohm $s^{-0.5}$), ω is corresponding angular frequency (rad s^{-1}), S_a is surface area of the active electrode (cm^2), n is valence of Zn^{2+}, F is Faraday's constant (C mol^{-1}), C is the molar concentration of Zn^{2+} (mol L^{-1}) in gel electrolyte. σ of $K_xV_2O_5{\cdot}nH_2O$-2 and $V_2O_5{\cdot}nH_2O$-2 Zn-TFBs can be fitted from $Re(Z)$ at a serial of ω. Since R, F (96485.3383 C mol^{-1}) is constant and T, S_a, n, C for two Zn-TFBs are the same, $\frac{R^2T^2}{2{S_a}^2n^4F^4C^2}$ can be seen as a coefficient K. Then, σ^2 is inversely proportional to $D_{zn^{2+}}$, so, knowing the comparative relationship of σ between two Zn-

TFBs enable to draw the comparative relation of $D_{Zn^{2+}}$ between them.

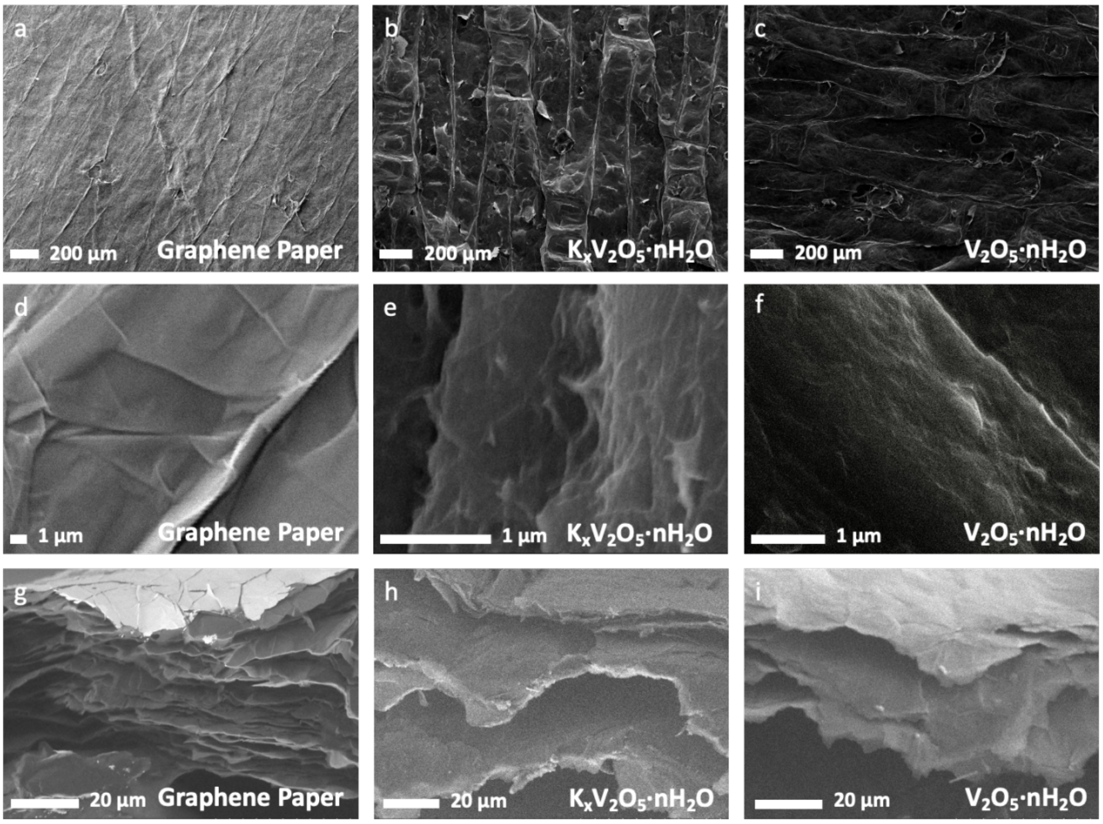

Figure S1. *SEM images for (a, d) graphene paper and electrodeposited (b, e) $K_xV_2O_5·nH_2O$, (c, f) $V_2O_5·nH_2O$ at different magnifications. SEM cross-sections of (g) bare graphene paepr and (h) $K_xV_2O_5·nH_2O$, (i) $V_2O_5·nH_2O$ electrodeposited on it.*

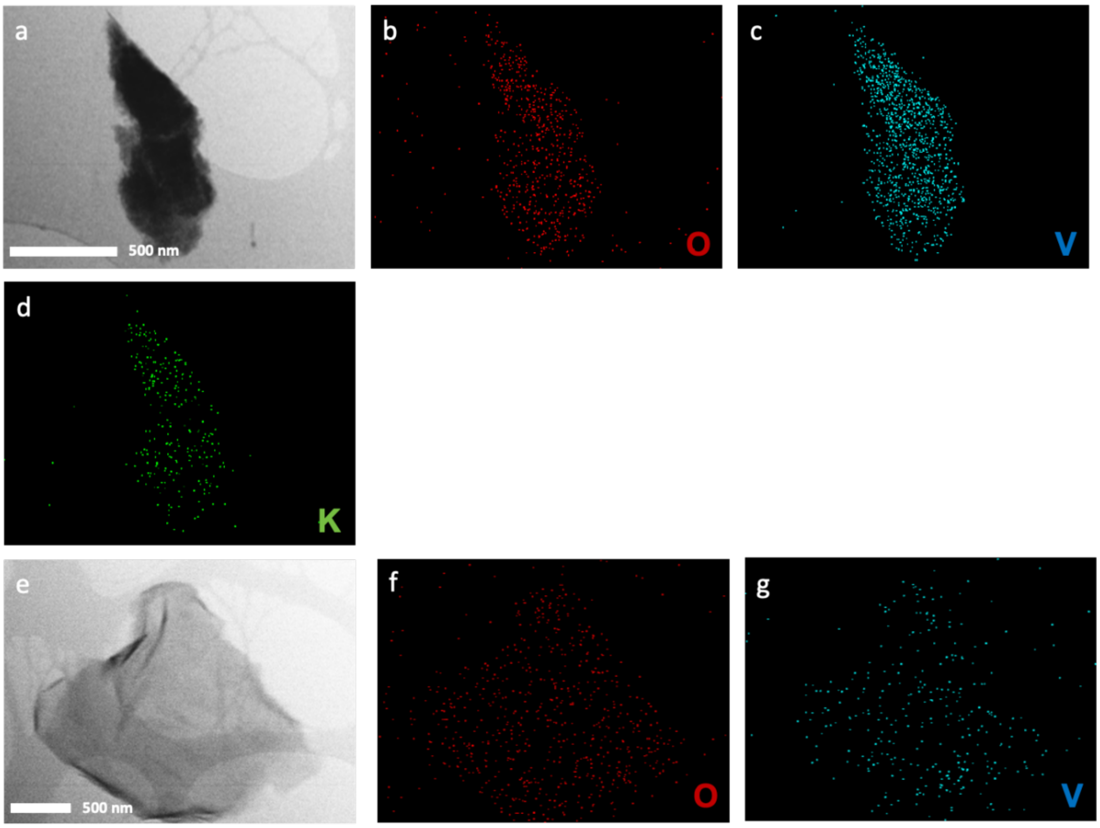

Figure S2. *The bright-field TEM image of (a) $K_xV_2O_5{\cdot}nH_2O$, (e) $V_2O_5{\cdot}nH_2O$, and corresponding element mapping of (b, f) oxygen, (c, g) vanadium and (d) potassium.*

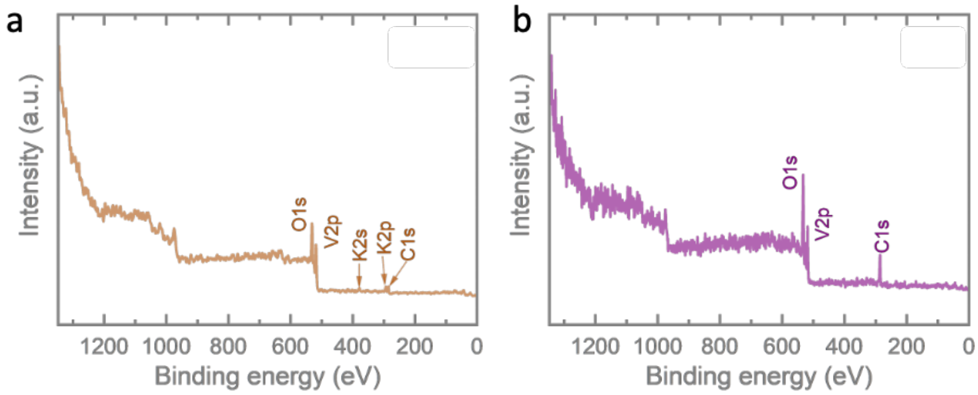

Figure S3. *XPS survey spectra of (a) $K_xV_2O_5{\cdot}nH_2O$ and (b) $V_2O_5{\cdot}nH_2O$.*

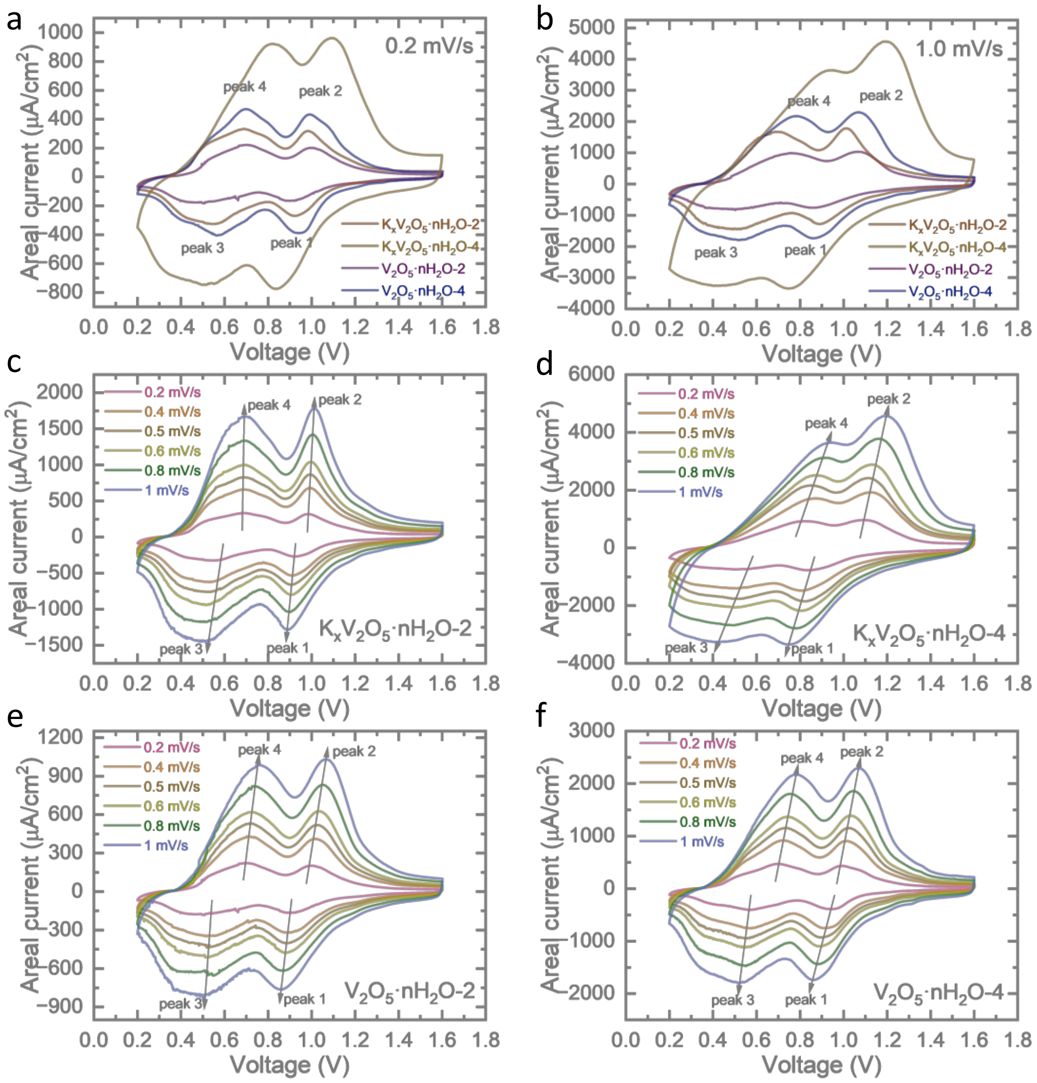

Figure S4. *Comparative CV graphs for V_2O_5·nH_2O-2, $K_xV_2O_5$·nH_2O-2, V_2O_5·nH_2O-4, and $K_xV_2O_5$·nH_2O-4 cells at (a) 0.2 mV s^{-1} and (b) 1.0 mV s^{-1}. CV graphs for (c) $K_xV_2O_5$·nH_2O-2, (d) $K_xV_2O_5$·nH_2O-4, (e) V_2O_5·nH_2O-2, (f) V_2O_5·nH_2O-4 cell at scan rates of 0.2, 0.4, 0.5, 0.6, 0.8, and 1.0 mV s^{-1}.*

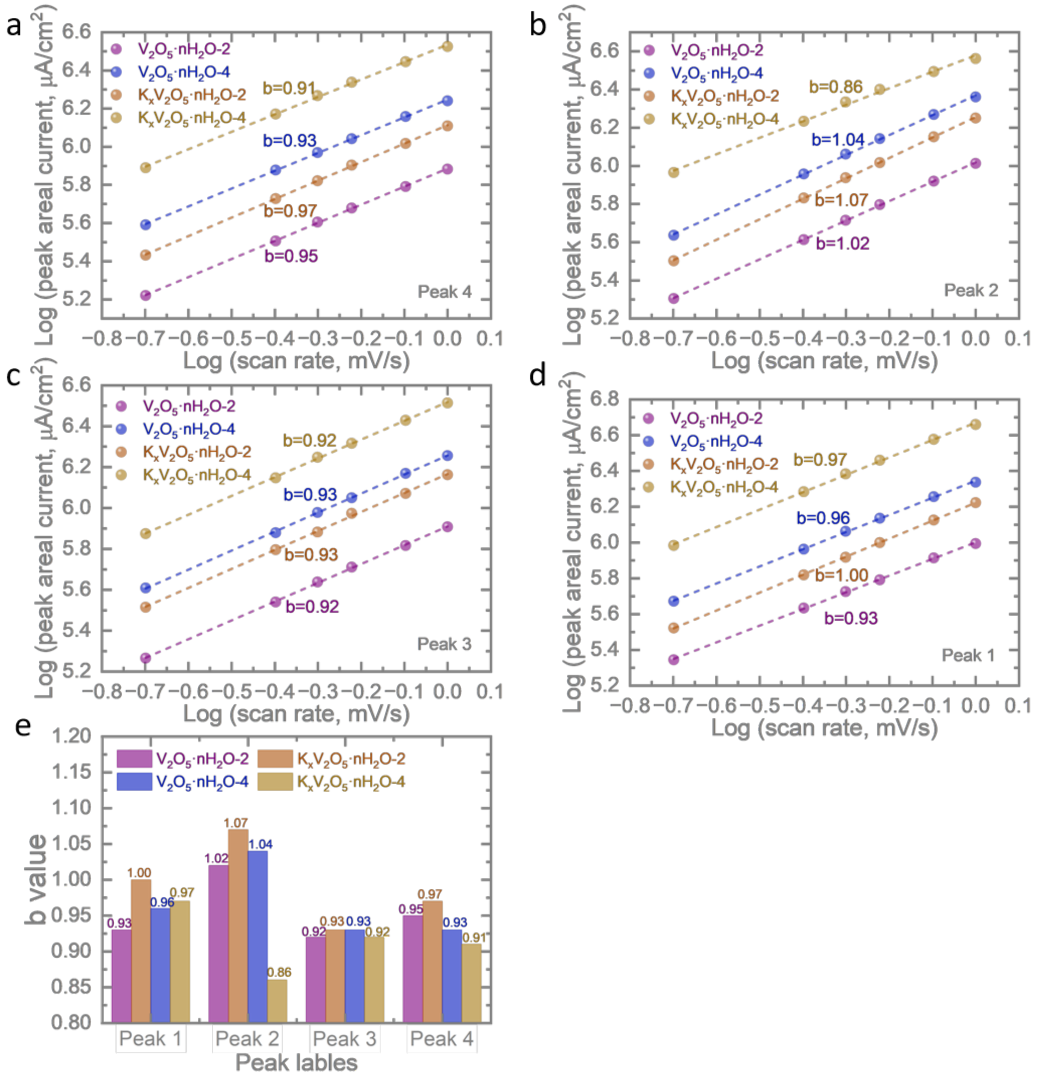

Figure S5. *The computational process of b values for $V_2O_5{\cdot}nH_2O$-2, $K_xV_2O_5{\cdot}nH_2O$-2, $V_2O_5{\cdot}nH_2O$-4, $K_xV_2O_5{\cdot}nH_2O$-4 cells at peak (a) 4, (b) 2, (c) 3, (d) 1 and their (e) conclusive results.*

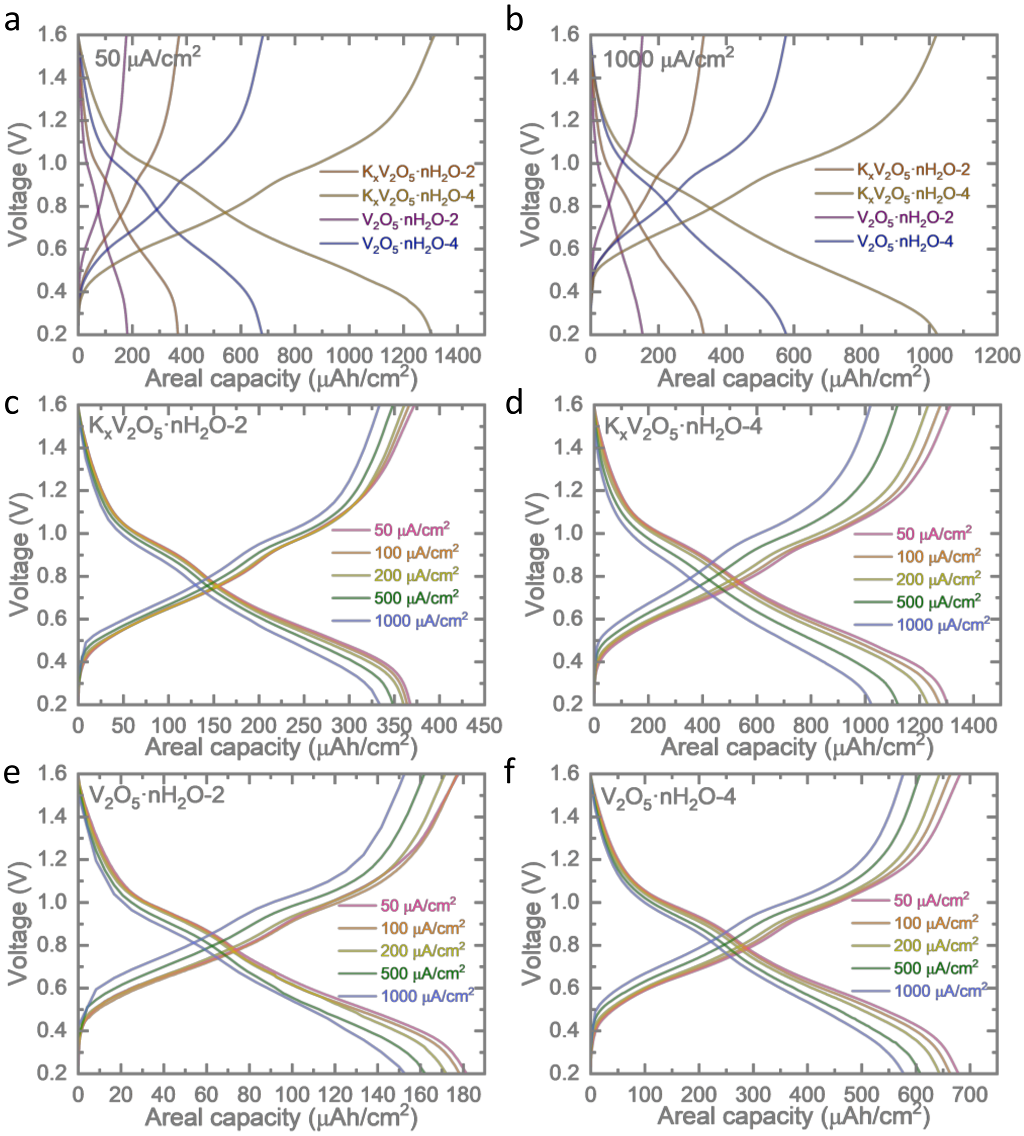

Figure S6. *Comparative GDC graphs for $K_xV_2O_5{\cdot}nH_2O$-2, $V_2O_5{\cdot}nH_2O$-2, $K_xV_2O_5{\cdot}nH_2O$-4 and $V_2O_5{\cdot}nH_2O$-4 cells at areal current of (a) 50 μA cm^{-2} and (b) 1000 μA cm^{-2}. GDC graphs for (c) $K_xV_2O_5{\cdot}nH_2O$-2, (d) $K_xV_2O_5{\cdot}nH_2O$-4, (e) $V_2O_5{\cdot}nH_2O$-2, (f) $V_2O_5{\cdot}nH_2O$-4 cells tested at areal currents of 50 μA cm^{-2}, 100 μA cm^{-2}, 200 μA cm^{-2}, 500 μA cm^{-2}, and 1000 μA cm^{-2}.*

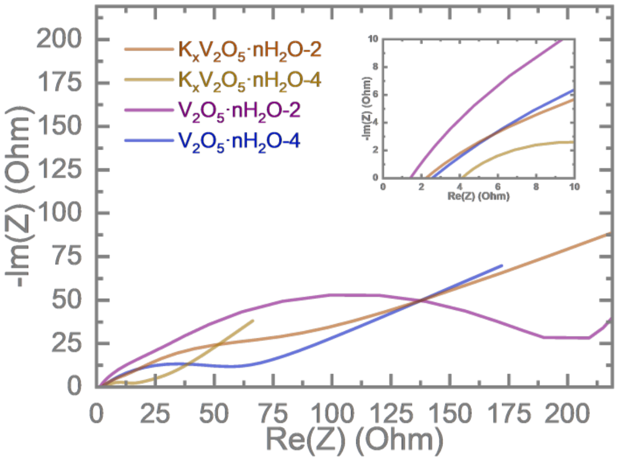

Figure S7. *Nyquist plot for $K_xV_2O_5·nH_2O$-2, $K_xV_2O_5·nH_2O$-4, $V_2O_5·nH_2O$-2, and $V_2O_5·nH_2O$-4 cells with aqueous 3M $Zn(CF_3SO_3)_2$ electrolyte.*

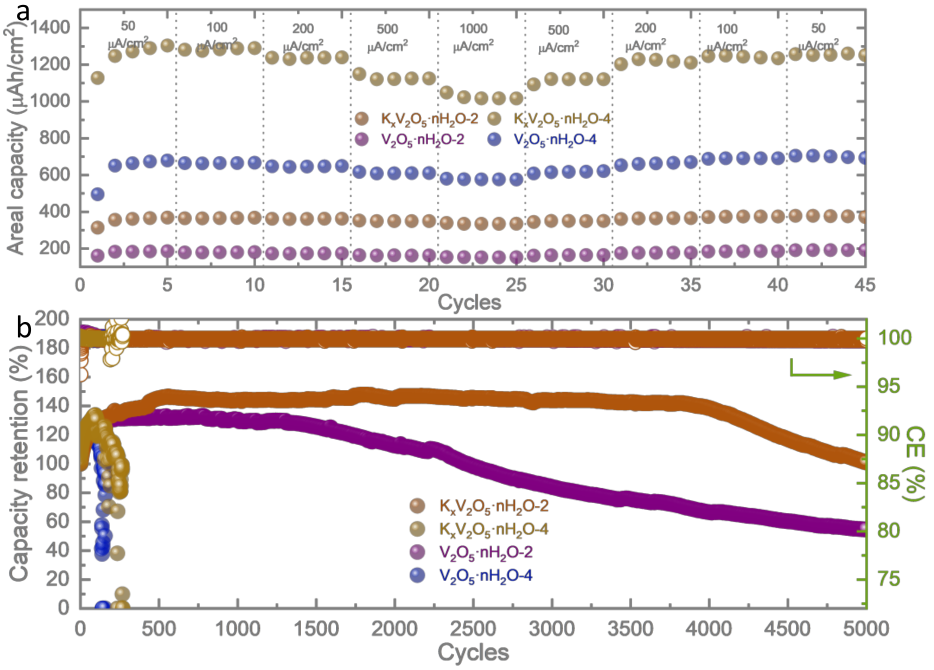

Figure S8. *(a) Rate test results for $K_xV_2O_5·nH_2O$-2, $V_2O_5·nH_2O$-2, $K_xV_2O_5·nH_2O$-4, and $V_2O_5·nH_2O$-4 coin cells at areal currents from 50 μA cm^{-2} to 1000 μA cm^{-2}, and (b) their cycle performances at 1000 μA cm^{-2}.*

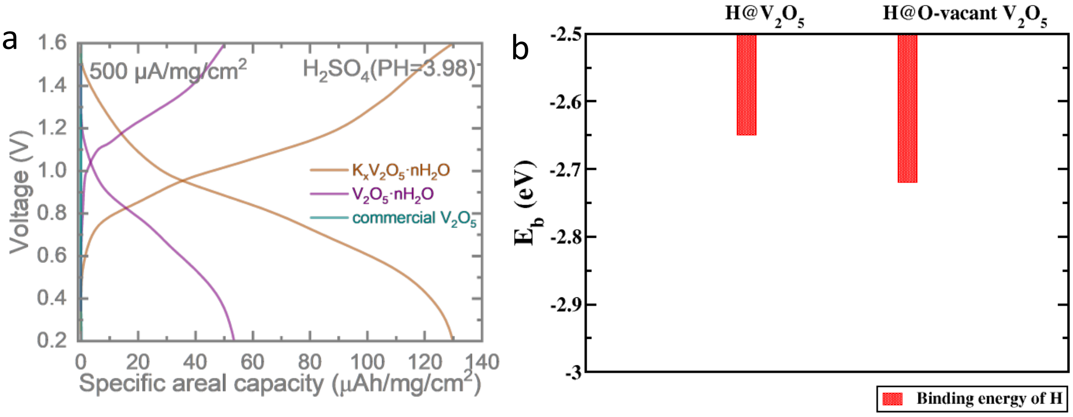

Figure S9. (a) Comparative GDC graphs for $K_xV_2O_5{\cdot}nH_2O$, $V_2O_5{\cdot}nH_2O$, commercial V_2O_5 cells with H_2SO_4 (pH = 3.98) as electrolyte at areal current of 500 µA mg^{-1} cm^{-2}. (b) Binding energy of H with the V_2O_5 and the O-vacant V_2O_5 samples.

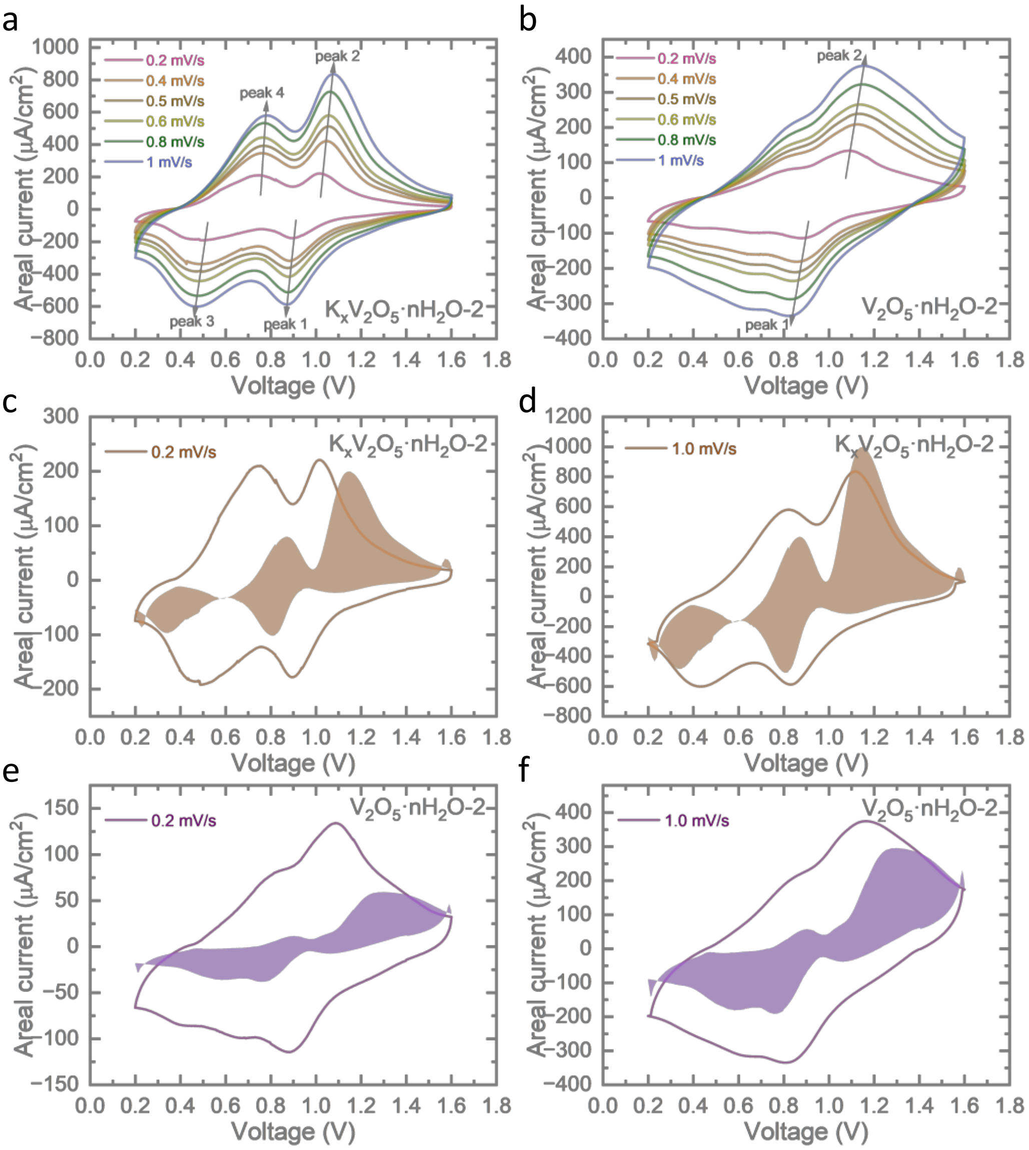

Figure S10. *CV graphs for (a) $K_xV_2O_5{\cdot}nH_2O$-2, (b) $V_2O_5{\cdot}nH_2O$-2 Zn-TFBs with GG gel electrolyte at scan rates of 0.2, 0.4, 0.5, 0.6, 0.8, and 1.0 mV s^{-1}. Estimation of capacitance-dominated behavior from CV profiles of (c, d) $K_xV_2O_5{\cdot}nH_2O$-2 and (e, f) $V_2O_5{\cdot}nH_2O$-2 at 0.2 and 1.0 mV s^{-1}, with the capacitive current contribution highlighted by the shaded regions.*

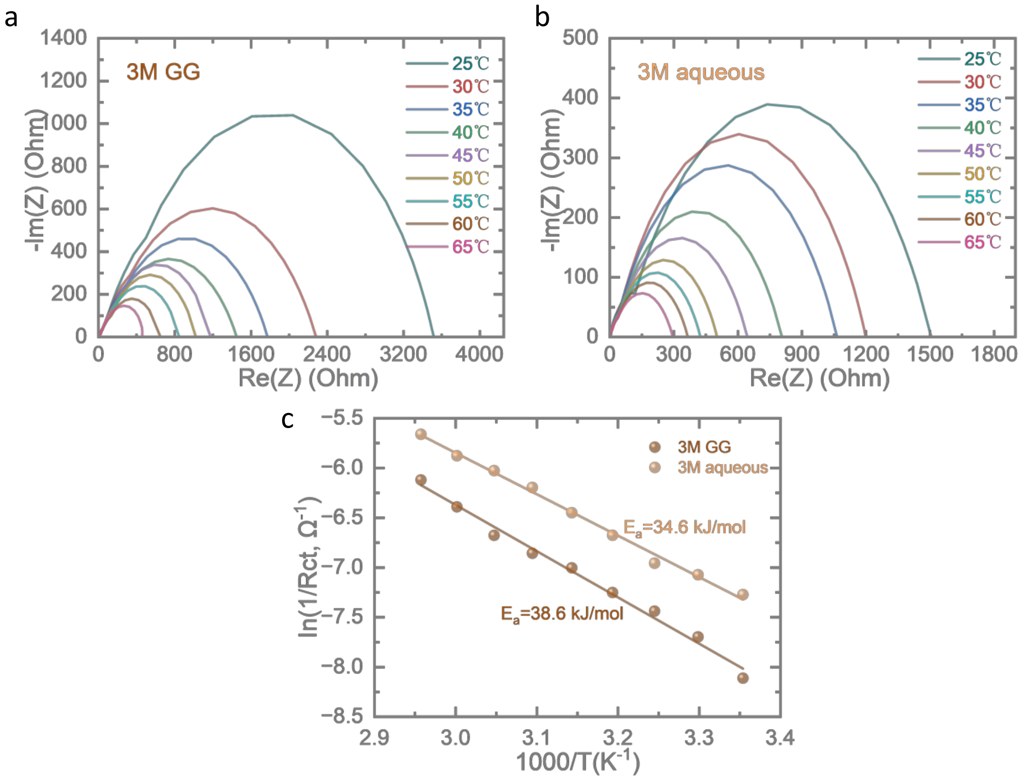

Figure S11. *Nyquist plots of Zn//Zn symmetrical coin cells using (a) GG gel electrolyte and (b) 3 M Zn(CF_3SO_3)$_2$ aqueous electrolyte at different temperatures, along with (c) the corresponding activation energy (E_a).*

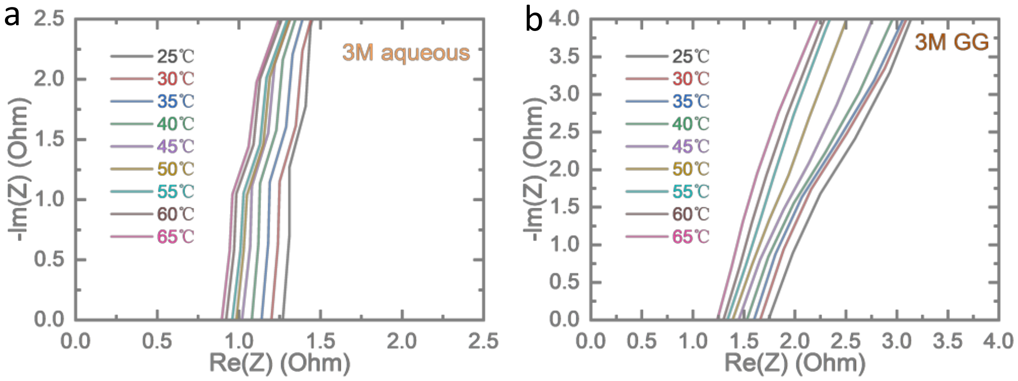

Figure S12. *Nyquist plots of SS//SS symmetrical coin cells with (a) 3 M Zn(CF_3SO_3)$_2$ aqueous electrolyte and (b) GG gel electrolyte.*

Table 1. *The ionic conductivity of aqueous 3M Zn(CF_3SO_3)$_2$ electrolyte at different temperatures.*

T (°C)	25	30	35	40	45	50	55	60	65
σ (mS cm-1)	13.31	14.10	14.80	15.71	16.61	17.14	17.59	18.29	18.85

Table 2. *The ionic conductivity of GG gel electrolyte at different temperatures.*

T (°C)	25	30	35	40	45	50	55	60	65
σ (mS cm-1)	9.71	10.17	10.56	11.02	11.62	12.19	12.64	12.96	13.68

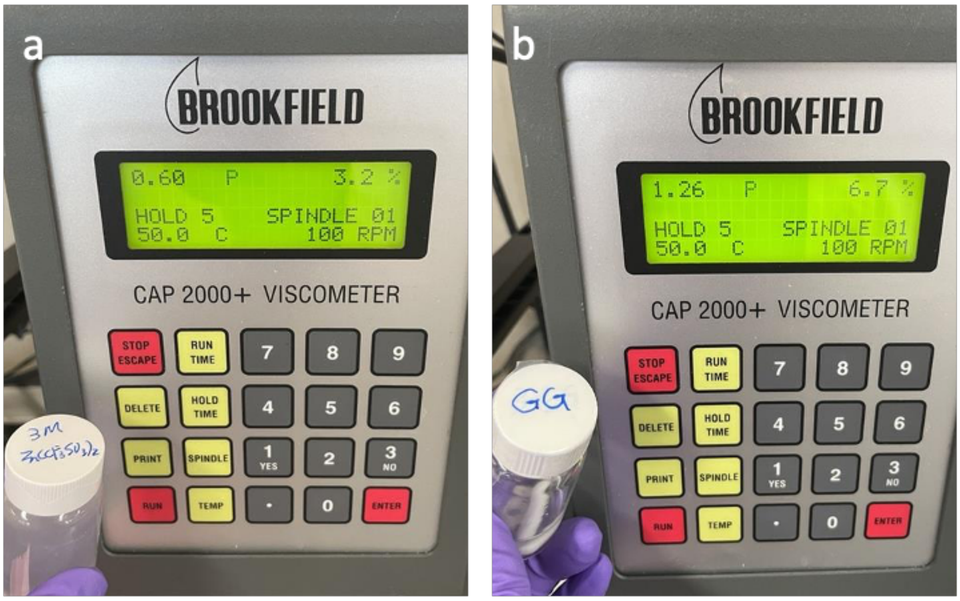

Figure S13. *Pictures showing the viscosity of (a) 3M Zn(CF_3SO_3)$_2$ aqueous electrolyte and (b) GG gel electrolyte tested at 50 ^{o}C.*

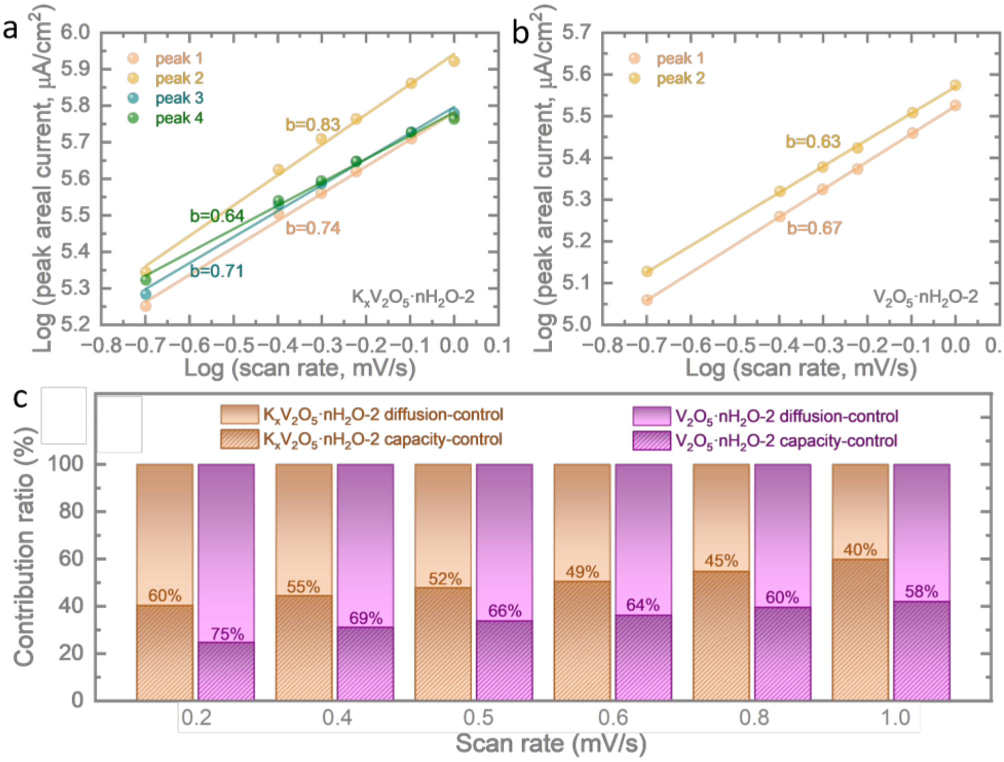

Figure S14. *b-value calculations for the respective redox peaks of (a) $K_xV_2O_5{\cdot}nH_2O$-2 and (b) $V_2O_5{\cdot}nH_2O$-2 Zn-TFBs with GG gel electrolyte. (c) Capacitive and diffusion-controlled contributions for $K_xV_2O_5{\cdot}nH_2O$-2 and $V_2O_5{\cdot}nH_2O$-2 at scan rates of 0.2, 0.4, 0.5, 0.6, 0.8, and 1.0 mV s^{-1}.*

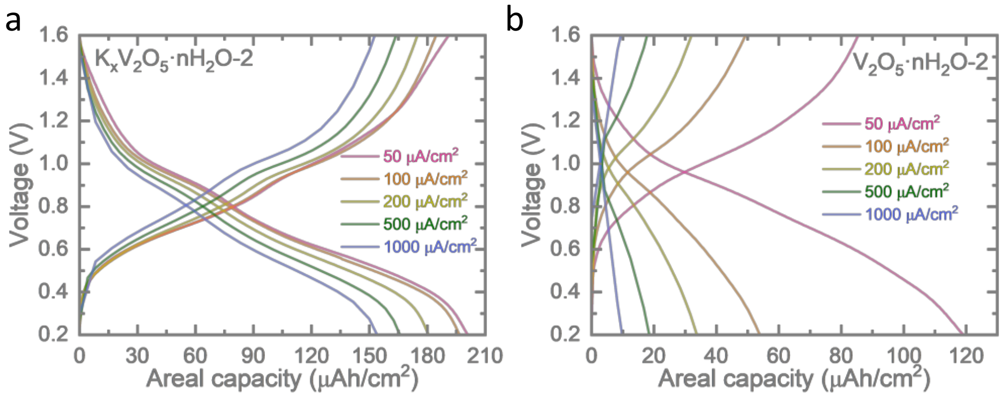

Figure S15. *GDC profiles of (a) $K_xV_2O_5{\cdot}nH_2O$-2 and (b) $V_2O_5{\cdot}nH_2O$-2 Zn-TFBs with GG gel electrolyte at areal current densities of 50, 100, 200, 500, and 1000 μA cm^{-2}.*

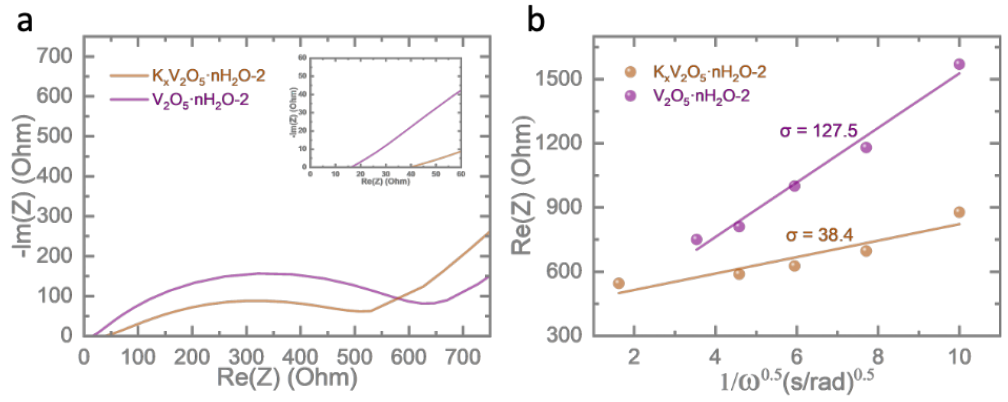

Figure S16. *(a) Nyquist plots of $K_xV_2O_5·nH_2O$-2 and $V_2O_5·nH_2O$-2 Zn-TFBs with GG gel electrolyte, and (b) the corresponding Zn^{2+} diffusion coefficients.*

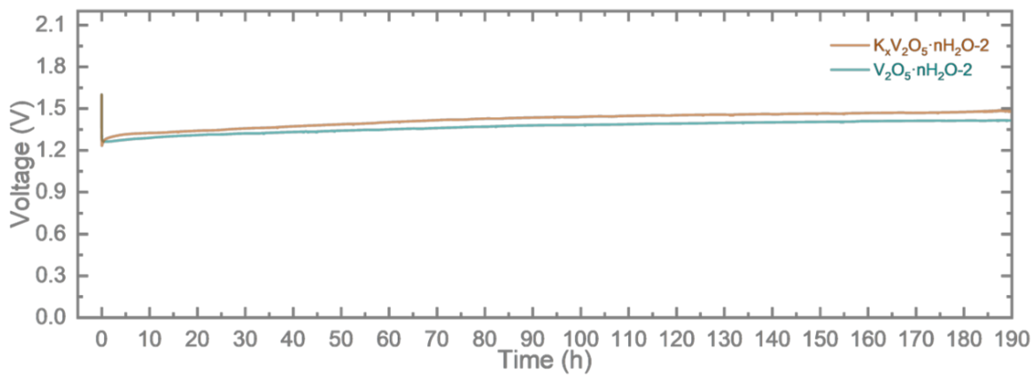

Figure S17. *Self-discharge behavior of $K_xV_2O_5·nH_2O$-2 and $V_2O_5·nH_2O$-2 Zn-TFBs with GG gel electrolyte.*

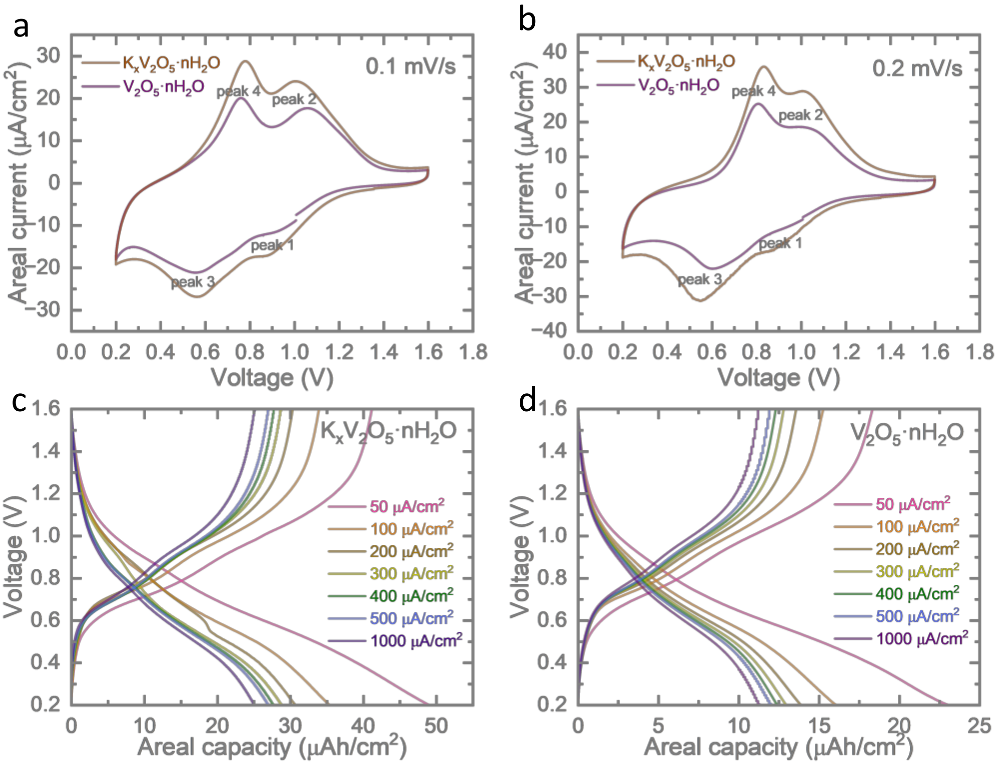

Figure S18. *Comparative CV curves of $K_xV_2O_5{\cdot}nH_2O$ and $V_2O_5{\cdot}nH_2O$ Zn-MBs at (a) 0.1 and (b) 0.2 mV s^{-1}. GDC profiles of (c) $K_xV_2O_5{\cdot}nH_2O$ and (d) $V_2O_5{\cdot}nH_2O$ Zn-MBs at areal currents of 50, 100, 200, 300, 400, 500, and 1000 μA cm^{-2}.*

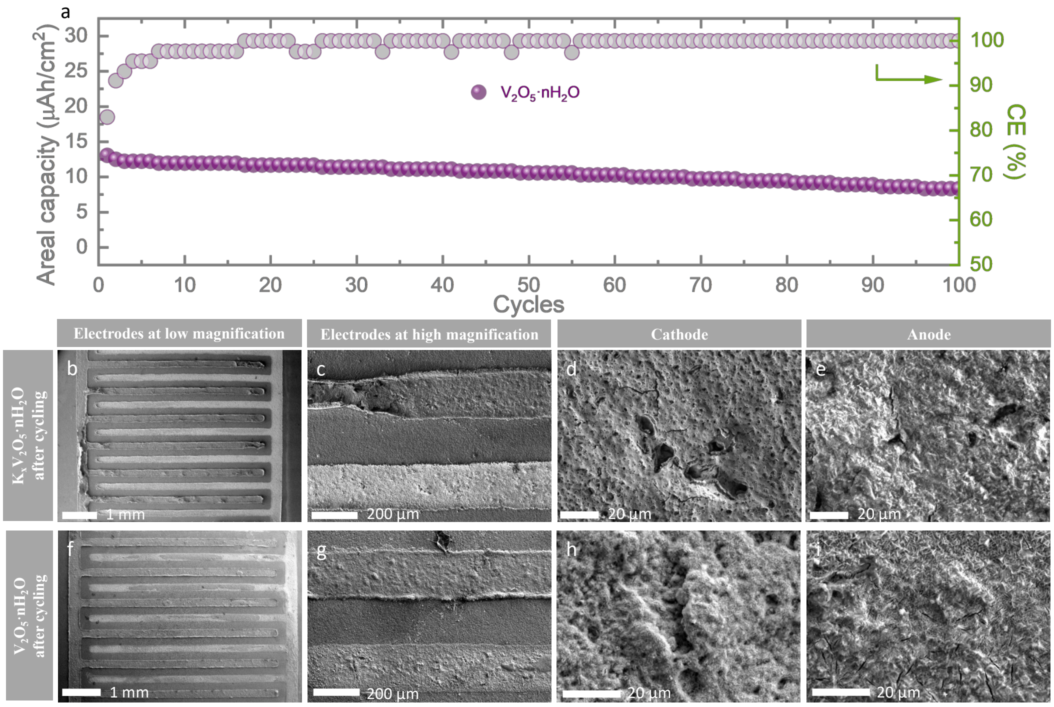

Figure S19. *(a) long-term cycling performance of $V_2O_5{\cdot}nH_2O$ Zn-MB at 500 μA cm^{-2}.*

SEM images of cycled Zn-MBs after 200 cycles: (b-e) $K_xV_2O_5 \cdot nH_2O$ and (f-i) $V_2O_5 \cdot nH_2O$ at different magnifications.

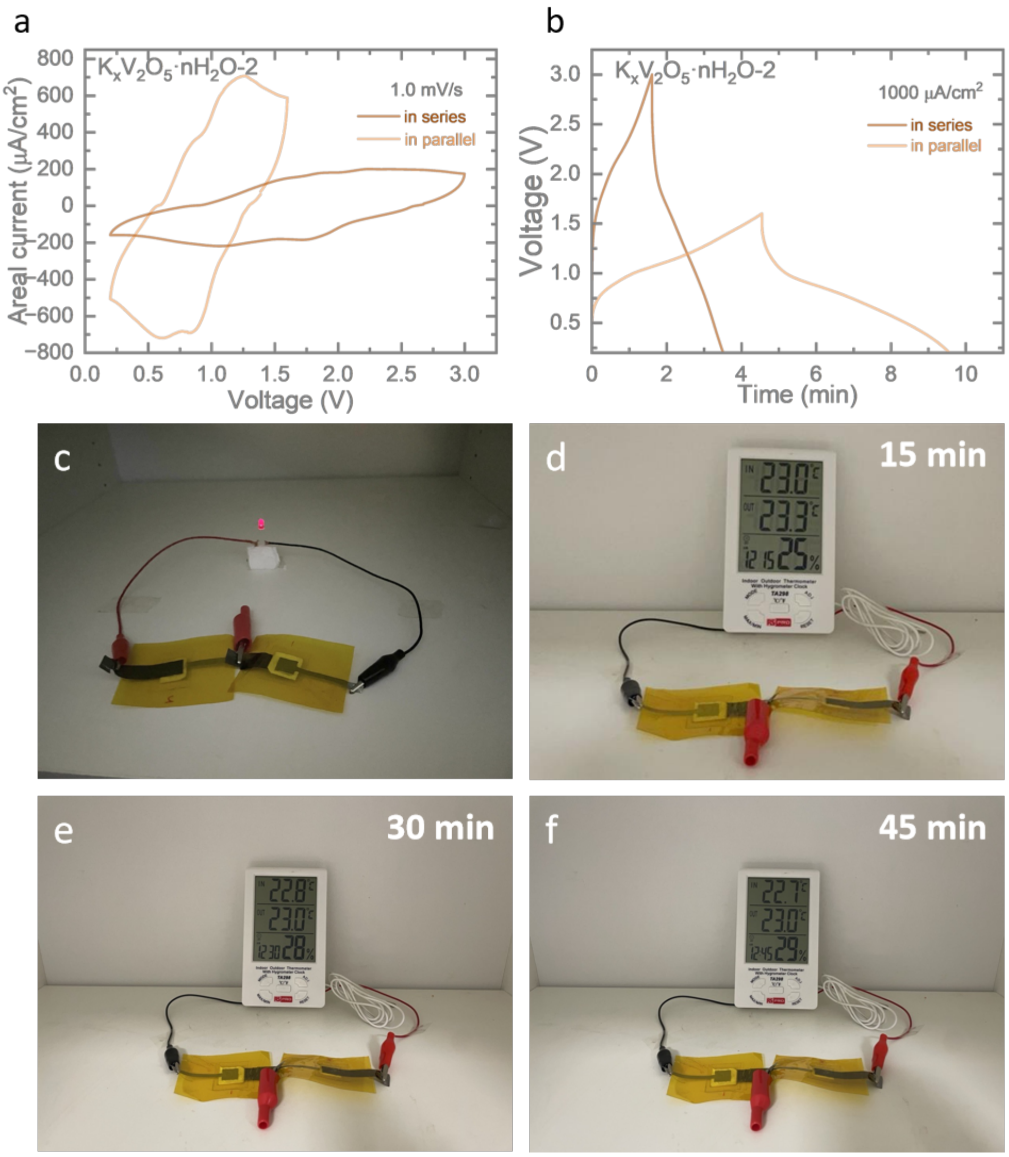

Figure S20. *(a) CV curves and (b) GCD profiles of $K_xV_2O_5 \cdot nH_2O$-2 Zn-TFBs connected in series and parallel. Demonstration of practical applications: (c) powering an LED and (d–f) driving an indoor/outdoor thermometer with hygrometer and clock using two planar $K_xV_2O_5 \cdot nH_2O$-2 Zn-TFBs connected in series.*

References

[1] L. J. Bartolotti, K. Flurchick, *Rev. Comput. Chem.* **1996**, *5*, 187–216.

[2] N. Argaman, G. Makov, *Am. J. Phys.* **2000**, *68*, 69–79.

[3] N. M. Harrison, *NATO Sci. Ser. Sub Ser. III Comput. Syst. Sci.* **2003**, *187*, 45–70.

[4] G. Kresse, J. Furthmüller, *Comput. Mater. Sci.* **1996**, *6*, 15–50.

[5] G. Kresse, J. Furthmüller, *Phys. Rev. B* **1996**, *54*, 11169–11186.

[6] J. P. Perdew, K. Burke, M. Ernzerhof, *Phys. Rev. Lett.* **1996**, *77*, 3865–3868.

[7] P. E. Blöchl, *Phys. Rev. B* **1994**, *50*, 17953–17979.

[8] S. Grimme, J. Antony, S. Ehrlich, H. Krieg, *J. Chem. Phys.* **2010**, *132*, 154104.
